# Childhood Transitions Between Weight Status Categories: Evidence from the UK Millennium Cohort Study

**DOI:** 10.1007/s40273-024-01361-3

**Published:** 2024-04-03

**Authors:** Olu Onyimadu, Nerys M. Astbury, Felix Achana, Stavros Petrou, Mara Violato

**Affiliations:** 1 Nuffield Department of Primary Care Health Sciences, University of Oxford, Radcliffe Observatory Quarter, Woodstock Road, Oxford, OX2 6GG UK; 2 Nuffield Department of Population Health, University of Oxford, Old Road Campus, Oxford, OX3 7LF UK

## Abstract

**Background:**

Assessing the cost-effectiveness of interventions targeting childhood excess weight requires estimates of the hazards of transitioning between weight status categories. Current estimates are based on studies characterized by insufficient sample sizes, a lack of national representativeness, and untested assumptions.

**Objectives:**

We sought to (1) estimate transition probabilities and hazard ratios for transitioning between childhood weight status categories, (2) test the validity of the underlying assumption in the literature that transitions between childhood bodyweight categories are time-homogeneous, (3) account for complex sampling procedures when deriving nationally representative transition estimates, and (4) explore the impact of child, maternal, and sociodemographic characteristics.

**Methods:**

We applied a multistate transition modeling approach accounting for complex survey design to UK Millennium Cohort Study (MCS) data to predict transition probabilities and hazard ratios for weight status movements for children aged 3–17. Surveys were conducted at ages 3 (wave 2 in 2004), 5 (wave 3 in 2006), 7 (wave 4 in 2008), 11 (wave 5 in 2012), 14 (wave 6 in 2015), and 17 (wave 7 in 2018) years. We derived datasets that included repeated body mass index measurements across waves after excluding multiple births and children with missing or implausible bodyweight records. To account for the stratified cluster sample design of the MCS, we incorporated survey weights and jackknife replicates of survey weights. Using a validation dataset from the MCS, we tested the validity of our models. Finally, we estimated the relationships between state transitions and child, maternal, and sociodemographic factors.

**Results:**

The datasets for our primary analysis consisted of 10,399 children for waves 2–3, 10,729 for waves 3–4, 9685 for waves 4–5, 8593 for waves 5–6, and 7085 for waves 6–7. All datasets consisted of roughly equal splits of boys and girls. Under the assumption of time-heterogeneous transition rates (our base-case model), younger children (ages 3–5 and 5–7 years) had significantly higher annual transition probabilities of moving from healthy weight to overweight (0.033, 95% confidence interval [CI] 0.026–0.041, and 0.027, 95% CI 0.021–0.033, respectively) compared to older children (0.015, 95% CI 0.012–0.018, at ages 7–11; 0.018, 95% CI 0.013–0.023, at ages 11–14; and 0.018, 95% CI 0.013–0.025 at ages 14–17 years). However, the resolution of unhealthy weight was more strongly age-dependent than transitions from healthy weight to non-healthy weight states. Transition hazards differed by child, maternal, and sociodemographic factors.

**Conclusions:**

Our models generated estimates of bodyweight status transitions in a representative UK childhood population. Compared to our scenario models (i.e., time-homogeneous transition rates), our base-case model fits the observed data best, indicating a non-time-homogeneous pattern in transitions between bodyweight categories during childhood. Transition hazards varied significantly by age and across subpopulations, suggesting that conducting subgroup-specific cost-effectiveness analyses of childhood weight management interventions will optimize decision-making.

**Supplementary Information:**

The online version contains supplementary material available at 10.1007/s40273-024-01361-3.

## Key Points for Decision Makers

**Table:** 

This multistate transition modeling study found a statistically significant age gradient, with children between 3 and 7 years showing a greater tendency to switch between healthy weight and overweight or obesity than older children.
Sex, ethnicity, gestational age, family income, and maternal factors were also estimated to predict transitions between bodyweight categories.
Our findings favor targeted approaches to childhood weight management rather than population-wide strategies since the estimated transition rates, which ultimately inform decision-analytic models designed to assess the cost-effectiveness of childhood excess weight interventions, differ across population subgroups.

## Introduction

Childhood excess weight, a collective term for childhood overweight and obesity, remains a significant public health concern in many countries due to its association with overweight and obesity in adulthood and premature mortality [1]. Globally, 6% of children under 5 years of age were overweight or obese in 2020 [2]. In England, between 2019/2020 and 2020/2021, the prevalence of obesity rose from 9.9 to 14.4% among children aged 4–5 years and from 21 to 25.5% among children aged 10–11 years [3].

Given the increasing prevalence of childhood excess weight and limited resources, the aspirations of governments and decision-makers include prioritizing the implementation of cost-effective strategies for the management of childhood excess weight [4]. Economic evaluations conducted alongside randomized controlled trials of interventions targeting childhood excess weight tend to assess costs and consequences only over short follow-up periods, missing long-term costs and benefits [5]. Decision-analytic models can address this limitation by estimating costs and consequences over extended time horizons [4, 6].

Decision-analytic models, in particular, state transition models, are the most frequently used vehicle for the conduct of economic evaluations of interventions targeting childhood excess weight [5]. State transition models characterize the movements between health states such as underweight, healthy weight, overweight, and obesity over discrete time steps [7], untangling the complex epidemiological patterns and stochastic processes surrounding chronic disease progression [6, 7].

Movements between health states over discrete time intervals are driven by transition estimates (probabilities or hazards), which may vary by the effect of interest. Repeated measurements in longitudinal studies allow for the estimation of transition probabilities between health states using a state transition modeling approach such as multistate modeling (MSM) [8–13]. In a continuous-time MSM framework (continuous-time Markov process), individuals observed over time move between a finite number of states in continuous time [7, 14–16], and the impact of sociodemographic characteristics and risk factors associated with the accumulation of childhood excess weight on bodyweight trajectories can be accounted for. The derived transition estimates can be incorporated into decision-analytic modeling-based economic evaluations of strategies targeting childhood excess weight.

Several methodological weaknesses in the application of continuous-time MSM have been observed, which potentially hinder the use of the derived transition parameters in health economic evaluations. For instance, transition probabilities have been assumed to be homogeneous throughout the course of childhood without justification or robust testing and validation [8–10, 12, 17]. Further, studies have been based on restricted sample sizes and age groups [8–10, 12] and unrepresentative samples [8, 9, 12].

In this study, we used the Millennium Cohort Study (MCS), a UK-representative sample of children aged 3–17 years, to develop Markov multistate transition models using time-to-event analysis. These models incorporated a stratified cluster sampling design to obtain transition estimates for bodyweight status. We tested the assumption of time-homogeneous transition probabilities and conducted internal validation of our estimates. We also examined the relationships between transition probabilities and factors that could plausibly be associated with transitions between bodyweight categories.

## Methods

### Study Design and Population

The MCS is a nationally representative longitudinal survey of around 19,000 children born in the UK between 2000 and 2002 [18]. To date, seven MCS waves have been conducted at ages 9 months (wave 1 in 2001), 3 years (wave 2 in 2004), 5 years (wave 3 in 2006), 7 years (wave 4 in 2008), 11 years (wave 5 in 2012), 14 years (wave 6 in 2015), and 17 years (wave 7 in 2018). Trained interviewers used standardized methodologies to measure child height and weight (to the nearest 0.1 cm and nearest 0.1 kg, respectively) and data on sociodemographic and family characteristics [19]. Our assessment of transitions between bodyweight categories spanned children aged 3 (when height was first measured) to 17 years. The datasets used for this study are publicly available and details for accessing them are provided in the electronic supplementary material (“Data availability” section).

The study population for our primary analysis included singletons with at least two body mass index (BMI) measurements across MCS waves and complete data on relevant variables (described below). BMI was calculated as kg/m^2^ and then converted into standard deviation (SD) scores (BMI z-scores) using the British Growth Reference (UK90) for age-adjusted and sex-specific categories [20]. Conversions to BMI z-scores were implemented using Stata 17 statistical software [21, 22]. Children in our study population were then grouped into bodyweight categories for each BMI record based on the UK cut-offs for population monitoring: less than or equal to the 2nd centile (underweight), greater than the 2nd and less than the 85th centile (healthy weight), greater than or equal to the 85th and less than the 95th centile (overweight), and greater than or equal to the 95th centile (obesity) [23]. In sensitivity analyses, we also applied the World Health Organization (WHO) references and classifications for children aged 60 months or younger (underweight [or thinness]: BMI z-score < −2; healthy weight: BMI z-score ≥ −2 and ≤ 2; overweight: BMI z-score > 2 and ≤ 3; and obesity: BMI z-score > 3) and children aged 61 months or older (underweight [or thinness]: BMI z-score < −2; healthy weight: BMI z-score ≥ −2 and ≤ 1; overweight: BMI z-score > 1 and ≤ 2; and obesity: BMI z-score > 2) [24]. This study followed the Strengthening the Reporting of Observational Studies in Epidemiology (STROBE) reporting guideline for cohort studies [26].

### Covariates

The choice of individual-level child, mother, and sociodemographic factors to include in our analysis was informed by the relevant literature [8–10, 12, 19, 27]. Child and mother factors were as follows: child's sex (male or female); ethnicity (white and non-white); gestational age at birth (preterm [< 37 weeks], early term [≥ 37 and < 39 weeks], full term [≥ 39 and < 41 weeks], late term [≥ 41 and < 42 weeks], and post-term [≥ 42 weeks]) [28]; mother’s age at birth of child (12–19, 20–29, and 30 plus years); mother’s BMI category during pregnancy (underweight, healthy weight, and overweight/obesity); mother’s frequency of alcohol consumption during pregnancy (monthly or more frequently, less than once a month, and never); and mode of delivery (normal, assisted, planned cesarean section, and emergency cesarean section). Sociodemographic factors were as follows: mother’s highest academic qualification (first/higher degree, diplomas in higher education, A/AS/S levels, O level/General Certificate of Secondary Education (GCSE) grades A–C, GCSE grades D–G, other academic qualifications, and none of these qualifications); and family income categorized as Organisation for Economic Co-operation and Development (OECD)-weighted quintiles [29].

### Statistical Analysis

#### Model Derivation and Validation

Our evaluation was based on a multistate transition modeling approach within the maximum-likelihood estimation framework specified by the MSM function [15] in the statistical software R [30]. Our multistate model aims to estimate transitions between bodyweight categories*.* It is defined by a continuous-time, finite-state stochastic process, with the Markovian assumption that subsequent movement to another state depends only on the current state and not on past states [14, 31]. The principal outputs from our Markov multistate transition model were transition hazard rates (also known as transition intensities), which represent the instantaneous risk of moving from one state to another, such as from healthy weight to overweight. Transition probabilities could then be derived for cycle lengths of interest (such as weeks/months/years) by taking the matrix exponential of the transition intensity matrix [15].

Our models specified four predefined BMI categories: underweight, healthy weight, overweight, and obesity. We allowed for only clinically plausible transition intensities between adjacent waves of data. For example, even if a child had a healthy weight status in wave 2 and obesity in wave 3, an instantaneous progression was only possible between adjacent states. Therefore, though unobserved, the child must have progressed to an overweight status first. This assumption is consistent with existing studies on childhood BMI transitions [9, 10]. Consequently, our four-state model consisted of six possible transitions: underweight to healthy weight; healthy weight to underweight; healthy weight to overweight; overweight to healthy weight; overweight to obesity; and obesity to overweight. The model structure is illustrated and further described in the electronic supplementary material (“Multistate model structure” section).

We estimated transition intensities and annual transition probabilities for a primary analysis (our base case) and two scenario analyses. The primary model reflected our hypothesis that transition probabilities are not time-homogeneous across all waves, i.e., they depend significantly on a child’s age in the year of observation. For the primary analysis, we extracted a dataset of children with measures of BMI for each wave. We then extracted five corresponding pairwise datasets from adjacent sequential waves as follows: waves 2 and 3 (ages 3 and 5), waves 3 and 4 (ages 5 and 7), waves 4 and 5 (ages 7 and 11), waves 5 and 6 (ages 11 and 14), and waves 6 and 7 (ages 14 and 17). Repeated measures of BMI were required to construct each pairwise dataset such that the same children in the earlier wave of a pair were followed into the later wave of the pair (see the electronic supplementary material: “Dataset extraction: primary analysis” section). A time-homogeneous process was assumed only within these five adjacent pairs of MCS waves with piecewise linkage between successive pairwise datasets. In other words, the transition hazards estimated were constant within pairwise datasets but varied between pairwise datasets (see the electronic supplementary material, “Dataset extraction: primary analysis” section, for details).

Scenarios 1 and 2 assumed that transition probabilities were time-homogeneous across all waves, i.e., throughout childhood. We extracted a single dataset for scenario 1 consisting of individuals with at least one repeated measure of BMI between wave 2 (age 3) and any other wave (see the electronic supplementary material: “Dataset extraction: scenario analyses” section). In scenario 2, we assumed that the transition probabilities between waves 2 and 3 were identical to those between successive pairs of adjacent waves. Therefore, for this scenario, we only extracted a dataset of children with repeated BMI measurements between waves 2 and 3 (ages 3 and 5).

Finally, we derived a validation dataset, which we used to test the performance of the three models by comparing observed and predicted (model) estimates of prevalence. We identified 5486 children with complete BMI measurements from waves 2 to 7, forming the basis of our validation dataset. Subsequently, we normalized and scaled the survey weights attached to each child a hundred times, replicating an additional 218,366 children. We merged the replicated datasets with the original validation dataset, which resulted in 221,436 children. Of these, 0.78%, 72.70%, 15.90%, and 10.63% fell in the underweight, healthy weight, overweight, and obesity categories at baseline (wave 2/age 3), respectively. These baseline percentages were then inputted as baseline cohorts into Markov traces of our derived base-case and scenario analyses to simulate the projected yearly prevalence for each bodyweight category. The validation process is further described in the electronic supplementary material (“Model validation” section), and estimates of weighted and unweighted prevalence by wave using both the UK cut-offs for population monitoring and the WHO cut-offs are reported in the electronic supplementary material (“Observed prevalence” section).

#### Integrating Complex Survey Design Within Models

The sample design of the MCS data aimed to ensure an adequate representation of the UK childhood population as well as guarantee sufficient sampling for critical subgroups [32]. Consequently, the survey was geographically clustered and disproportionately stratified with oversampling of children in disadvantaged socioeconomic circumstances and, in England, children from minority ethnic backgrounds. Despite these measures, the MCS surveys reported complex response patterns characterized by higher non-response rates for ethnic minorities and families in deprived areas and inter-wave attrition [18, 33]. The MCS database constructors provide survey weights that account for the clustered sample design, the unequal probability of being sampled, survey nonresponse, and adjust for inter-wave attrition [18]. To avoid bias in the estimation of point estimates (transition hazards and probabilities), we incorporated these survey weights into a validated adaption of the MSM R package [34].

Confidence intervals derived without adequate handling of variance will be artificially narrow [34]. Variance estimation for parameter point estimates posed an additional layer of complexity to our estimation strategy because the survey design variables provided by the MCS database constructors are suited to the linearization/Taylor series estimation method [35], which does not lend itself to the MSM function. The computationally intensive replication method we adopted is an alternative to linearization [36]. We derived 398 jackknife (jk-n) replicate weights (matching the number of clusters in the MCS as stipulated in the methods for variance calculation by Valliant and Dever [37]) in Stata [21] for each modeling dataset and then used the replicated weights to calculate estimates of variance [37–39]. We accelerated the time-consuming variance estimation process using parallel computing approaches [40]. Technical details of the steps taken in our estimation of replicate weights are described in the electronic supplementary material (“Incorporating complex survey design” section).

#### Robustness Check for Complete Case Analysis

Similar to other longitudinal studies of childhood excess weight using the MCS, where maximum likelihood estimation formed the basis of the statistical method [41, 42] or where other methods such as regression analyses were applied [27, 43], our analyses were based on complete-case analysis, where we included only children with complete data on the child, mother, and sociodemographic variables, outlined earlier. We tested the robustness of the derived transition hazards from our primary analysis by estimating and comparing transition hazards before and after excluding children with incomplete data on explanatory variables. Our sensitivity analysis entailed the estimation of unadjusted transition hazards before excluding children with incomplete data on the child, mother, and sociodemographic variables.

## Results

After the exclusion of children with missing or implausible bodyweight records and/or missing relevant variables, our primary analysis consisted of 10,399 children for the waves 2–3 dataset, 10,729 for the waves 3–4 dataset, 9685 children for the waves 4–5 dataset, 8593 children for the waves 5–6 dataset, and 7085 children for the waves 6–7 dataset. A detailed description of the data extraction process and the percentage of missingness for covariates is provided in the electronic supplementary material (“Dataset extraction: primary analysis” section). Baseline and sociodemographic characteristics for waves 2–3 are reported in Table 1.

**Table 1:** Descriptive statistics for the base-case dataset for waves 2–3 (*n* = 10,399)

Variable	Variable levels	*n*	%
Mean (SD) age in years at wave 2			3.13 (0.20)
Sex			
	Boys	5,227	50.26
	Girls	5172	49.74
Distribution of BMI category^a^			
	Underweight	126	1.21
	Healthy weight	7331	70.5
	Overweight	1718	16.52
	Obesity	1224	11.77
Ethnicity			
	White	9115	87.65
	Non-white	1284	12.35
Gestational age at birth			
	Preterm (<37 weeks)	696	6.69
	Early term (≥37 and <39 weeks)	1928	18.54
	Full term (≥39 and <41 weeks)	5198	49.99
	Late term (≥41 and <42 weeks)	2202	21.18
	Postterm (≥42 weeks)	375	3.61
Mothers' age at birth of child			
	12 to 19 years	680	6.54
	20 to 29 years	4599	44.23
	30 years and over	5120	49.24
Mother BMI category during pregnancy			
	Underweight	535	5.14
	Healthy weight	6821	65.59
	Overweight or obesity	3043	29.26
Mothers' frequency of alcohol consumption during pregnancy			
	Monthly or more frequent	1767	16.99
	Less than once a month	1514	14.56
	Never	7118	68.45
Mode of delivery			
	Normal	7036	67.66
	Assisted	1109	10.66
	Planned cesarean section	957	9.2
	Emergency cesarean section	1297	12.47
Mothers' highest academic qualification at baseline			
	First/higher degree	2000	19
	Diplomas in higher education	1035	10
	A /AS /S levels	1083	10.41
	O level/GCSE grades A-C	3611	35
	GCSE grades D-G	1015	9.76
	Other academic qualifications	222	2.13
	None of these qualifications	1433	13.78
Family income: OECD weighted quintiles at baseline (age 9 months)			
	Lowest quintile	1874	18.02
	Second quintile	2158	20.75
	Third quintile	2119	20.38
	Fourth quintile	2188	21.04
	Highest quintile	2060	19.81

*BMI* body mass index, *GCSE* General Certificate of Secondary Education, *OECD* Organisation for Economic Co-operation and Development

^a^The estimated BMI distributions are based on British 1990 growth reference (UK90) and UK cut-offs for population monitoring. These distributions have not been adjusted with survey weights

**Fig. 1:**
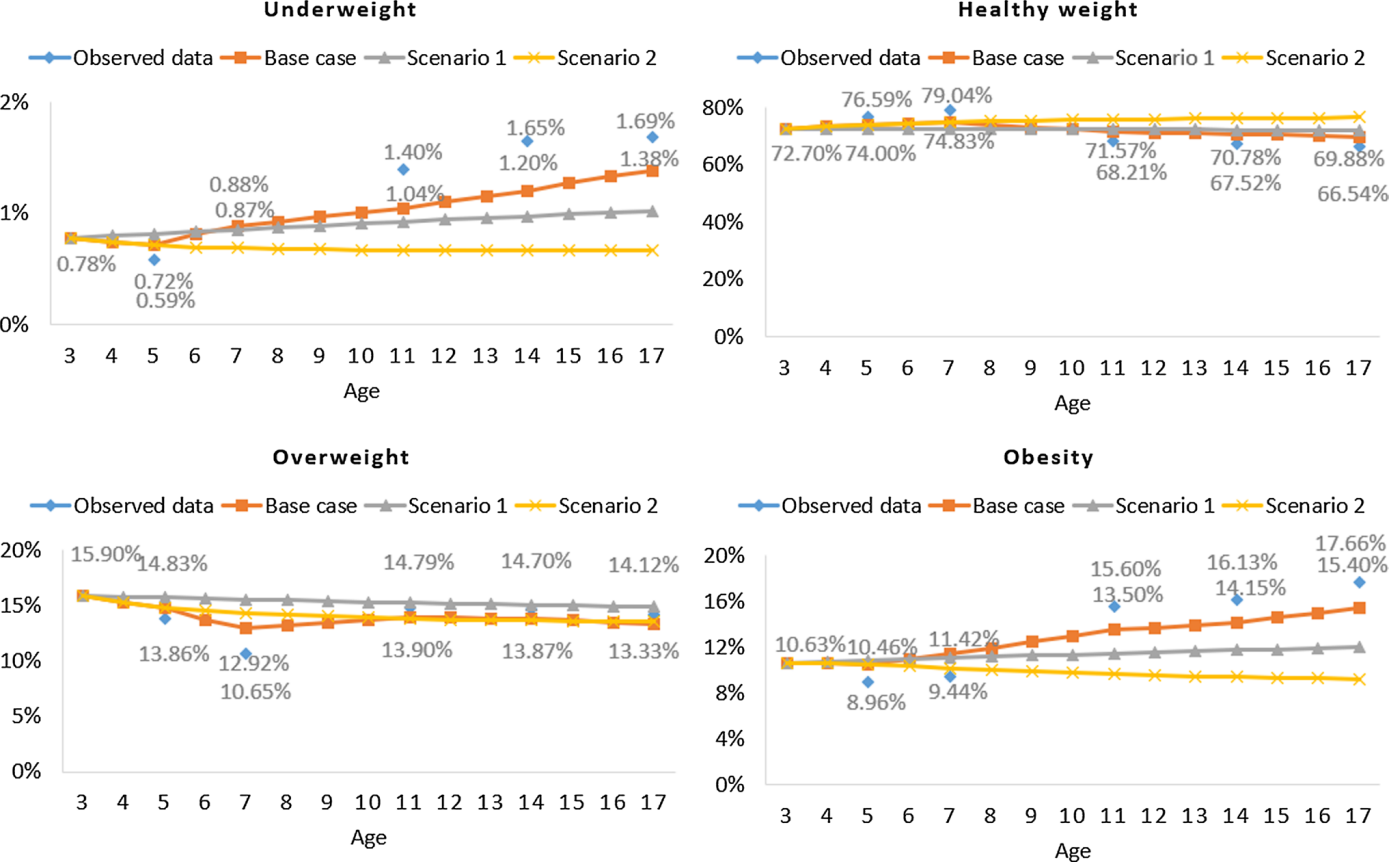
Observed and modeled projections for survey-weighted children followed from age 3 to 17 years

Figure 1 depicts the predicted and the observed prevalence in the validation dataset at six time points, signifying BMI measurements at waves 2, 3, 4, 5, 6, and 7. Prevalence estimates and their confidence intervals are tabulated in the electronic supplementary material (“Predicted-to-observed prevalence ratios” section). Figure 1 suggests that a non-time-homogenous pattern in transitions between bodyweight categories during childhood fits the observed data best. In sensitivity analysis, our replication of Fig. 1 using the WHO references and cut-offs (see the electronic supplementary material: “Sensitivity analyses” section, Figure 3) highlights a similar pattern. Prevalence estimates and their confidence intervals using the WHO references and cut-offs are also reported in the electronic supplementary material (“Sensitivity analyses” section, Table 6).

For comparison, we present the unadjusted transition hazards before excluding children with incomplete data on the child, mother, and sociodemographic variables (see the electronic supplementary material: “Sensitivity analysis” section, Table 7) alongside transition hazards for our primary analysis/complete case analysis (see the electronic supplementary material: “Sensitivity analysis” section, Table 8) in our robustness check. The relevant comparison is between transition hazard point estimates of corresponding datasets, not the confidence intervals, since we did not derive replicate weights for this sensitivity analysis. As previously noted in Sect. 2.3.2, the derivation and integration of replicate weights into variance estimation is computationally intensive. However, the point estimates of transition hazards estimated in both the sensitivity analysis and the primary analysis are unbiased because we applied the survey weights provided by MCS to account for the clustered sample design, the unequal probability of being sampled, and sample attrition. Examination of point estimates is sufficient to assess the effect of excluding children with missing data from our primary analysis. The similarity between the point estimates derived from the sensitivity and primary analyses is reassuring, as they lead to the same conclusions.

**Fig. 2:**
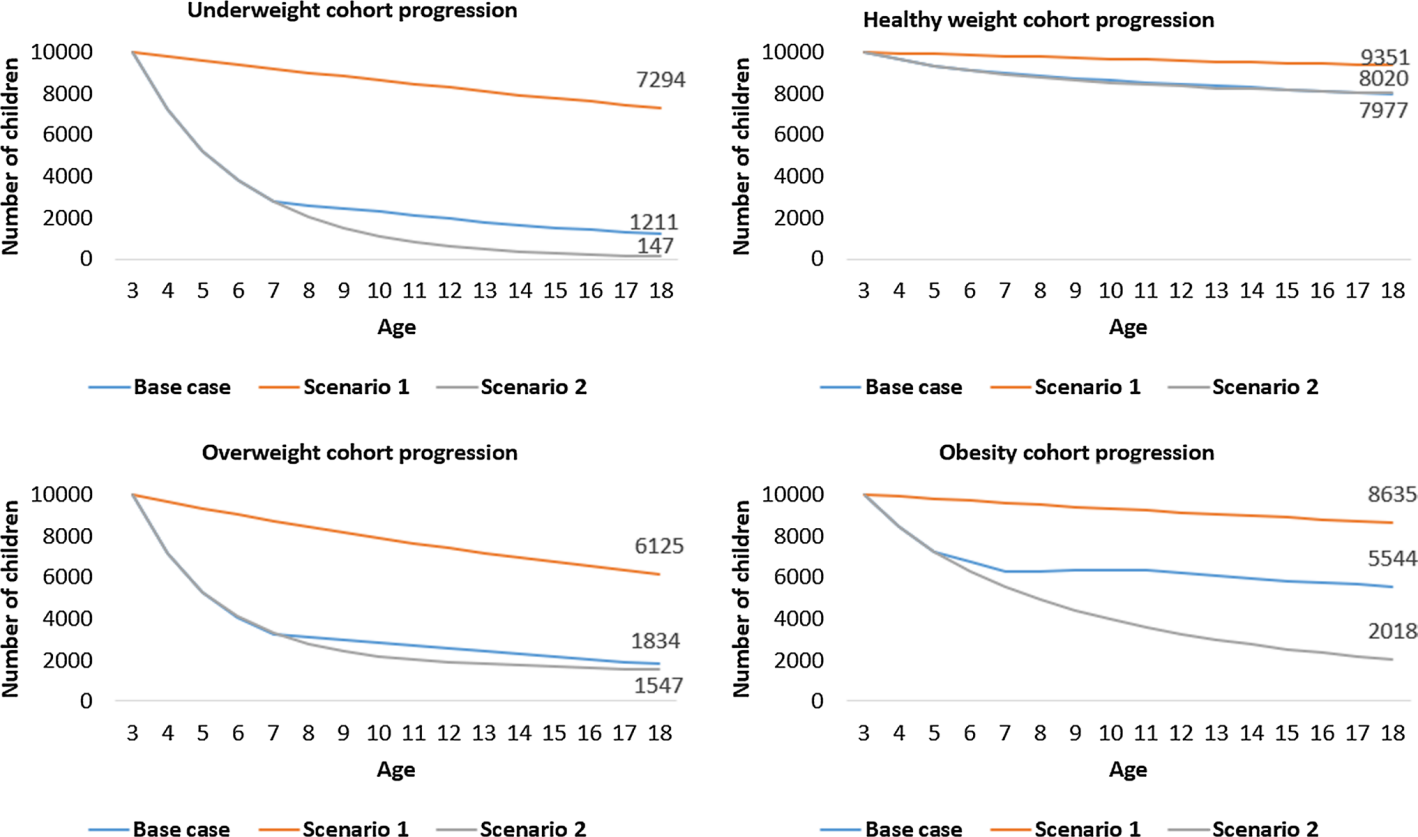
Modeled prevalence for four hypothetical cohorts of 10,000 children aged 3 at baseline in underweight, healthy weight, overweight, and obesity categories

The base-case estimates of annual transition probabilities across childhood and adolescence (Table 2) demonstrate a substantial age gradient in transitions from healthy weight to non-healthy weight states, with children over 7 years less likely to transition. However, the resolution of unhealthy weight is more strongly age-dependent, with children aged 3–7 more likely to transition from underweight, overweight, and obesity to healthy weight than older children. Our sensitivity analysis estimates of transition probabilities using the WHO references and cut-offs (see the electronic supplementary material: “Sensitivity analyses” section, Table 9) support these findings. In Fig. 2 (replicated using WHO references and cut-offs in the electronic supplementary material: “Sensitivity analyses” section, Fig. 4), we illustrate the consequence of choice of transition probabilities using four hypothetical cohorts of children. These homogeneous cohorts mirror many clinical trials targeting childhood obesity where baseline health or weight status profiles are similar within the trial arms [44–51]. However, the model can also assess a cohort of children with mixed baseline weight status profiles, as seen in Fig. 1. Considering the healthy weight cohort (top right quadrant, Fig. 2), for instance, by age 18, 79.77% of children in the UK with healthy weight at 3 years of age would still be in a state of healthy weight, given usual practice. The remaining 20.23% of the cohort would have transitioned into other health states over a 15-year period, as determined by the transition probabilities in Table 2.

**Table 2:** Primary analysis: unadjusted estimates of annual transition probabilities for transitions between weight status categories

To	Underweight		Healthy weight		Overweight		Obesity	
From								
	Annual probability	95% CI	Annual probability	95% CI	Annual probability	95% CI	Annual probability	95% CI
Waves 2 (mean age 3) to 3 (mean age 5), *n* = 10,399								
Underweight	0.7228	0.4704–0.8699	0.2719	0.1283–0.5155	0.0051	0.0018–0.0133	0.0002	0, 0.0008
Healthy weight	0.0024	0.001–0.0052	0.9624	0.9496–0.9719	0.0332	0.0261–0.0414	0.0020	0.001, 0.0038
Overweight	0.0003	0.0001–0.0008	0.1928	0.1534–0.2381	0.7132	0.6343–0.779	0.0937	0.0675, 0.1268
Obesity	0.0000	0–0.0001	0.0178	0.0101–0.0309	0.1419	0.1079–0.1817	0.8403	0.7873, 0.882
Waves 3 (mean age 5) to 4 (mean age 7), *n* = 10,729								
Underweight	0.7224	0.3939–0.8931	0.2733	0.1057–0.5929	0.0041	0.0012–0.0124	0.0002	0, 0.0008
Healthy weight	0.0039	0.0018–0.0073	0.9677	0.9566–0.9761	0.0267	0.0212–0.0331	0.0017	0.0009, 0.003
Overweight	0.0004	0.0001–0.001	0.1736	0.1392–0.2133	0.7281	0.6545–0.7893	0.0979	0.0714, 0.1312
Obesity	0.0000	0–0.0001	0.0097	0.0051–0.0177	0.0874	0.0616–0.1214	0.9029	0.8608, 0.9333
Waves 4 (mean age 7) to 5 (mean age 11), *n* = 9685								
Underweight	0.9326	0.8339–0.9735	0.0669	0.0263–0.1645	0.0005	0.0002–0.0016	0.0000	0, 0
Healthy weight	0.0014	0.0006–0.003	0.9836	0.9785–0.9873	0.0146	0.0119–0.0178	0.0004	0.0002, 0.0007
Overweight	0.0000	0–0.0001	0.0253	0.0155–0.0408	0.9223	0.8911–0.9444	0.0524	0.0401, 0.068
Obesity	0.0000	0–0	0.0002	0.0001–0.0006	0.0168	0.0097–0.0287	0.9830	0.9707, 0.9902
Waves 5 (mean age 11) to 6 (mean age 14), *n* = 8593								
Underweight	0.9042	0.7654–0.9628	0.0949	0.0369–0.2316	0.0009	0.0003–0.0029	0.0000	0, 0.0001
Healthy weight	0.0022	0.0009–0.0051	0.9798	0.971–0.9856	0.0175	0.0133–0.0229	0.0005	0.0002, 0.001
Overweight	0.0002	0–0.0003	0.0758	0.055–0.1034	0.8716	0.8185–0.9101	0.0524	0.0349, 0.0778
Obesity	0.0000	0–0	0.0016	0.0008–0.0033	0.0382	0.0258–0.0556	0.9602	0.9411, 0.9734
Waves 6 (mean age 14) to 7 (mean age 17) *n* = 7085								
Underweight	0.9186	0.8185, 0.9648	0.0806	0.035–0.1789	0.0008	0.0002–0.0025	0.0000	0, 0.0001
Healthy weight	0.0023	0.0006, 0.0079	0.9787	0.9654–0.9859	0.0183	0.0132–0.0249	0.0007	0.0003, 0.0018
Overweight	0.0001	0, 0.0005	0.0787	0.0551–0.1101	0.8507	0.7702–0.9043	0.0705	0.0406, 0.1192
Obesity	0.0000	0, 0	0.0017	0.0007–0.0041	0.0391	0.0245–0.061	0.9592	0.9349, 0.9748

Point estimates incorporate survey weights, and CIs were derived using replicate weights

*CI* confidence interval

The substantial sample sizes for sex subgroups allowed moderately narrow confidence intervals to be estimated for all primary analysis datasets. Transition hazard ratios and 95% confidence intervals for boys aged 3–17 (with girls as the reference category) are reported in Table 3. A lack of statistically significant difference between boys and girls is indicated if the confidence interval includes 1. Boys and girls showed significant differences in transition rates between weight status categories at all age groupings.

**Table 3:** Primary analysis: hazard ratios of transitions between weight status categories for sex for ages 3–17 years

Transition	Estimate	Lower 95% CI	Upper 95% CI
Waves 2–3 (ages 3–5 years): boys versus girls			
Underweight to healthy weight	0.8527	0.8370	0.8686
Healthy weight to underweight	1.7301	1.0978	2.7267
Healthy weight to overweight	1.2321	1.1844	1.2817
Overweight to healthy weight	0.8715	0.8448	0.8990
Overweight to obesity	1.1510	1.0717	1.2363
Obesity to overweight	0.9776	0.9708	0.9845
Waves 3–4 (ages 5–7 years): boys versus girls			
Underweight to healthy weight	1.0396	0.8370	1.2914
Healthy weight to underweight	1.0099	0.8084	1.2617
Healthy weight to overweight	1.0782	1.0393	1.1185
Overweight to healthy weight	1.1589	1.1487	1.1691
Overweight to obesity	1.1723	1.0963	1.2535
Obesity to overweight	1.1416	0.9959	1.3087
Waves 4–5 (ages 7–11 years): boys versus girls			
Underweight to healthy weight	1.9113	1.3044	2.8006
Healthy weight to underweight	1.0898	0.7912	1.5011
Healthy weight to overweight	1.1812	1.1651	1.1974
Overweight to healthy weight	1.2480	1.0584	1.4716
Overweight to obesity	0.9835	0.9418	1.0271
Obesity to overweight	1.1861	1.1150	1.2617
Waves 5–6 (ages 11–14 years): boys versus girls			
Underweight to healthy weight	0.3744	0.1797	0.7803
Healthy weight to underweight	1.2660	0.7591	2.1114
Healthy weight to overweight	0.7131	0.5899	0.8619
Overweight to healthy weight	1.3487	1.2386	1.4687
Overweight to obesity	0.9109	0.8675	0.9565
Obesity to overweight	1.1002	0.9808	1.2341
Waves 6–7 (ages 14–17 years): boys versus girls			
Underweight to healthy weight	0.6151	0.5517	0.6857
Healthy weight to underweight	1.7408	1.1573	2.6186
Healthy weight to overweight	1.0099	0.9960	1.0241
Overweight to healthy weight	1.0224	0.8992	1.1626
Overweight to obesity	1.7784	1.1780	2.6847
Obesity to overweight	1.4793	1.3940	1.5699

Reference category: girls

*CI* confidence interval

Transition hazard ratios for explanatory variables for children between 3 and 5 years are reported in the electronic supplementary material (“Hazard ratios for explanatory variables” section). The difference between levels of a variable and the referent level is determined to be statistically significant if the confidence interval does not include 1. Ethnicity, gestational age, mother’s BMI category during pregnancy, mother’s highest academic qualification, and family income had statistically significant adverse associations with hazards of transitioning between weight categories—non-white children were less likely to transition from underweight to healthy weight than their white counterparts. Preterm births were associated with higher likelihoods of transitioning from healthy weight to underweight compared to early and late-term births. Children born to mothers in the underweight category were more likely to progress from healthy weight to underweight (reference category: healthy weight mothers). Children born to mothers who were overweight or obese were more likely to transition from healthy weight to overweight and from overweight to obesity (reference category: healthy weight mothers). Using degree-level education as the referent, we did not find statistically significant differences in the hazards of children born to mothers in the “diplomas” and “other” academic qualifications categories. However, we found statistically significant differences in transition hazards for children born in the remaining categories, with the most significant differences in the “none” category. Children born to mothers with no academic qualifications were more likely to transition from healthy weight to overweight and obesity. Compared to families in the highest quintile of household income, children in all other quintiles experienced unfavorable transitions.

In terms of point estimates, children born to mothers aged 20–29 years and 30 years and over were less likely to progress from underweight to healthy weight (reference category: mother ≤ 19 years). However, this finding was not statistically significant. Mother’s consumption of alcohol during pregnancy and mode of delivery appeared not to play any significant role in a child’s chance of transitioning from one weight status to another for children aged 3–5 years.

## Discussion

### Summary of Main Findings

To our knowledge, our study is the first to incorporate a complex survey design in estimating transition hazard rates and confidence intervals between weight status categories in the UK childhood population. We developed, validated, and applied a multistate transition model that estimates transition rates between childhood bodyweight categories using data from the UK MCS. Compared to the scenario models, which assumed that transition probabilities were time-homogeneous across all waves, the base-case model showed closer agreement with the validation dataset for all states/bodyweight categories. Our primary analysis points to a substantial decrease in the prevalence of children in the healthy weight category with increasing age and concomitant increases in the prevalence of children in both the underweight and obese categories. We also found a substantial age gradient, with children between 3 and 7 years old showing a greater predisposition to switching between healthy weight and overweight or obese than older children. Sex, ethnicity, family income, and maternal factors also drove weight status transitions.

### Comparison with the Literature

In terms of prevalence, our findings are consistent with results from the National Child Measurement Programme (NCMP) [3], a nationally representative cross-sectional survey that assesses overweight and obesity levels in children living in England aged 4–5 years (reception class) and 10–11 years (year 6). The NCMP reported a higher prevalence of obesity and underweight in 10- to 11-year-olds compared to 4- to 5-year-olds from 2021 to 2022 [3]. Similar findings are highlighted in Fig. 1.

Point estimates and confidence intervals from earlier multistate transition studies based on surveys are likely biased because they do not account for survey weights and the unequal probability of selecting individuals [10]. Moreira et al. investigated bodyweight transitions in a Portuguese birth cohort of 4887 children, with BMI measurements taken at ages 4, 7, and 10 [10]. However, their analysis was not based on a nationally representative study population and did not validate results in the relevant population. Other continuous-time MSM studies relied on small sample sizes (507 [9], 928 [12], 1653 [8], and 2334 children [17]) that were not representative of the population from which they were drawn. These gaps constrain the robustness of comparisons with estimates from our analyses.

Our methodological approach to estimating transition rates differed fundamentally from previous studies, where transition rates and probabilities were estimated based on the assumption that variations and trends in estimates across waves were insignificant. The authors of a US-based study of 1653 children with 11 consecutive BMI assessments between the ages of 5 and 12 years reported that weight status tended to persist over time, implying a negligible probability of transitioning between weight status categories [8]. Such an assumption may be reasonable when considering a restricted age group and inevitable if sample sizes are limited or lack national representativeness. We tested this underlying assumption and found it implausible over the 14-year span of our study data. We, therefore, recommend that the premise of time-homogeneous transition rates should be limited only to adjacent waves of surveys to achieve the best fit to observed or population-wide data.

Compared with previous studies, our study investigated a broader range of sociodemographic characteristics and risk factors associated with childhood weight status transitions, with previous studies mainly focusing on transitions in weight status by sex, ethnicity and socioeconomic status and finding that they did matter [8, 9, 12, 17]. While further research is needed to determine the causal mechanisms that mediate these variations in transition, ignoring subgroup analyses when conducting cost-effectiveness analyses of interventions targeting childhood excess weight may bias policy recommendations.

### Strengths and Limitations

Multistate transition modeling is a compelling instrument for investigating epidemiological patterns in childhood excess weight and quantifying them in terms of transition probabilities and hazard ratios. Our results indicate that prevention and treatment interventions could have different outcomes in terms of cost-effectiveness, depending on the child’s age and subgroup at implementation, assuming other components of economic evaluations, such as costs, do not vary.

Our study benefits from the large sample size of the MCS and its longer time horizon (3–17 years) compared with previous studies, where the maximum follow-up was 8 years [9]. Our study provides for extrapolations of transition probabilities for any cycle length up to 18 years and incorporates the survey weights and complex design of the MCS, hence producing estimates that can be applied to modeling in the UK childhood population. A further strength is the robust testing of the prior methodological assumption regarding the time homogeneity of transition rates associated with childhood weight status categories. We justified our base-case model and showed that its results best fit the observed data.

Our study was not without limitations. Firstly, as with most longitudinal surveys, bodyweight status was interval censored between adjacent surveys, i.e., the exact time of transition is often unknown. In some instances, when estimating hazard rates for explanatory variables, the model estimates did not converge when there were no observed transitions within the data (see the electronic supplementary material: “Checking for model convergence” section). This limitation necessitated merging affected levels for specific covariates such as ethnicity, thus failing to provide the sufficient granularity required to investigate all sub-groups of interest. We recommend applying a half-cycle correction [52] when using our estimates of transition to design cost-effectiveness models.

Secondly, the intervals between waves were uneven. Since the assumption of time-homogeneous transition hazard rates does not hold throughout childhood, it then follows that estimates of annual transition probabilities are less accurate for adjacent waves with wider time intervals. Based on the overall model fitness for the data, we recommend that, at a minimum, future cohort studies collect biennial measurements of BMI during childhood.

Further, we note that there have been temporal changes in the prevalence of weight status categories, particularly during and since the coronavirus disease 2019 (COVID-19) pandemic. In the absence of new data, it is unclear what impact these changes have on the magnitude of effect for transition probabilities. However, our analyses provide the best estimates of state transitions given data constraints, and future updates can be made supported by the methodological approach in this study.

We acknowledge that our model estimates are better suited for cost-effectiveness modeling within a state transition, Markov framework. State transition models are currently the most widely applied approaches for conducting economic evaluations of childhood excess weight interventions [5]. Markov models are not as data-intensive as microsimulation models, such as discrete event simulation models. The consequence is that detail-oriented microsimulation models maximize the individual-level information provided by rich databases such as the MCS, thereby robustly expressing the heterogeneity of the sample [4]. There is an opportunity to extend the MCS analysis by replicating our study using microsimulation models.

The choice of reference population or standard for assessing growth and cut-off points for defining weight status category is worth careful consideration and justification because the estimates of prevalence and transition probabilities between bodyweight profiles are bound to differ when different standards and cut-offs are applied. For instance, using the WHO references and cut-offs, children in wave 2 (3-year-olds) are less likely to be classified under the overweight and obesity categories. Given its global perspective, the WHO’s cautious approach in classifying children under 5 is partly motivated to prevent the imposition of restrictive diets on young children [53]. We adopted the British 1990 growth reference (UK90) and UK cut-offs for population monitoring, used for most published studies on obesity and overweight among children in the UK in our primary analysis [23], and replicated several analyses using the WHO references and cut-offs [24]. The trends and patterns we identified were broadly consistent using both standards and cut-offs, as the findings based on the UK cut-offs are reinforced by sensitivity analyses using the WHO cut-offs.

## Conclusion

We generated transition estimates of bodyweight status in a representative UK childhood population. Our study demonstrated marked age-related gradients in the transitions between childhood bodyweight profiles, with children between the ages of 3 and 7 years more likely to move between healthy weight and excess weight than older children. The propensity for movements between weight status categories also varied by sex across all ages and was dependent on child, maternal, and sociodemographic factors. Transition estimates derived in this study can serve as input parameters in the cost-effectiveness modeling of interventions targeting childhood excess weight.

### Supplementary Information

Below is the link to the electronic supplementary material.

Supplementary file1 (DOCX 288 KB)

## Declarations

### Data Availability

Millennium Cohort Study (MCS) datasets are publicly available via the UK Data Service (https://beta.ukdataservice.ac.uk/datacatalogue/series/series?id=2000031#!/access-data), and further information about the MCS can be found at https://cls.ucl.ac.uk/cls-studies/millennium-cohort-study/. The seven datasets we used for our study can be found under the “GN 33359 Millennium Cohort Study – Survey and Biomeasures Data” Access data Tab at https://beta.ukdataservice.ac.uk/datacatalogue/series/series?id=2000031#!/access-data. On the UK Data Service website, these datasets are titled as follows: Millennium Cohort Study: Age 9 months, Sweep 1, 2001 (SN 4683), Millennium Cohort Study: Age 3, Sweep 2, 2004 (SN 5350), Millennium Cohort Study: Age 5, Sweep 3, 2006 (SN 5795), Millennium Cohort Study: Age 7, Sweep 4, 2008 (SN 6411), Millennium Cohort Study: Age 11, Sweep 5, 2012 (SN 7464), Millennium Cohort Study: Age 14, Sweep 6, 2015 (SN 8156), and Millennium Cohort Study: Age 17, Sweep 7, 2018 (SN 8682). The R package for fitting and predicting multistate model estimates is described in detail and can be downloaded from the paper by Jackson titled Multistate Modelling With R: The MSM Package (https://cran.r-project.org/web/packages/msm/vignettes/msm-manual.pdf). The adaption of the R code from this package we used to incorporate survey replicate weights can be found in the study by Brouwer et al. titled Transitions between cigarette, ENDS and dual use in adults in the PATH study (waves 1–4): multistate transition modeling accounting for complex survey design (https://tobaccocontrol.bmj.com/content/31/3/424).
