# Supplementary material for: Childhood Transitions Between Weight Status Categories: Evidence from the UK Millennium Cohort Study
Source: Pharmacoeconomics. 2024 Apr 3;42(6):649–61. doi: 10.1007/s40273-024-01361-3 (PMC11126508; doi:10.1007/s40273-024-01361-3)
Supplement: Supplementary file 1 — Supplementary file1 (DOCX 288 KB) [file 40273_2024_1361_MOESM1_ESM.docx]

# Supplementary material

**Accompanying Manuscript Title**: Childhood transitions between weight status categories: Evidence from the UK Millennium Cohort Study

**Authors:**

Olu Onyimadu, MSc^1*^ Nerys M Astbury, PhD^1^ Felix Achana, PhD^1^ Stavros Petrou, PhD^1#^ Mara Violato, PhD^2#^

1 Nuffield Department of Primary Care Health Sciences, University of Oxford, Radcliffe Observatory Quarter, Woodstock Road, Oxford OX2 6GG, UK; [olu.onyimadu@phc.ox.ac.uk](mailto:olu.onyimadu@phc.ox.ac.uk) (OO); [nerys.astbury@phc.ox.ac.uk](mailto:nerys.astbury@phc.ox.ac.uk) (NMA); [felix.achana@phc.ox.ac.uk](mailto:felix.achana@phc.ox.ac.uk) (FA); [Stavros.petrou@phc.ox.ac.uk](mailto:Stavros.petrou@phc.ox.ac.uk) (SP)

2 Nuffield Department of Population Health, University of Oxford, Old Road Campus, Oxford OX3 7LF, UK; [mara.violato@dph.ox.ac.uk](mailto:mara.violato@dph.ox.ac.uk) (MV)

* Correspondence: [olu.onyimadu@phc.ox.ac.uk](mailto:olu.onyimadu@phc.ox.ac.uk) (OO)

# Joint senior authors

1 Data availability

Millennium Cohort Study (MCS) datasets are publicly available via the UK Data Service (<https://beta.ukdataservice.ac.uk/datacatalogue/series/series?id=2000031#!/access-data>) and further information about the MCS can be found at <https://cls.ucl.ac.uk/cls-studies/millennium-cohort-study/>. The seven datasets we used for our study can be found under the “GN 33359 Millennium Cohort Study – Survey and Biomeasures Data” Access data Tab at <https://beta.ukdataservice.ac.uk/datacatalogue/series/series?id=2000031#!/access-data>. On the UK Data Service website, these datasets are titled: Millennium Cohort Study: Age 9 months, Sweep 1, 2001 (SN 4683), Millennium Cohort Study: Age 3, Sweep 2, 2004 (SN 5350), Millennium Cohort Study: Age 5, Sweep 3, 2006 (SN 5795), Millennium Cohort Study: Age 7, Sweep 4, 2008 (SN 6411), Millennium Cohort Study: Age 11, Sweep 5, 2012 (SN 7464), Millennium Cohort Study: Age 14, Sweep 6, 2015 (SN 8156), and Millennium Cohort Study: Age 17, Sweep 7, 2018 (SN 8682).

# 2 Multistate model structure


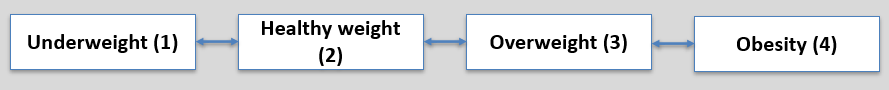


Figure 1 Model structure


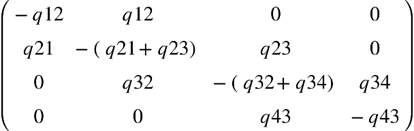


Figure 2 Matrix of transition intensities

Figure 1 and Figure 2 are diagrammatic and mathematical representations of the model structure, respectively, depicting multistate modeling in continuous time. The arrows in Figure 1 show what instantaneous hazards are allowed based on expert and clinical opinion. In this model, the instantaneous hazard of transitioning is only possible between adjacent health states (weight status categories).

In Figure 1, if 1, 2, 3, and 4 represent the health states underweight, healthy weight, overweight, and obesity, then q^12^, q^21^, q^23^, q^32^, q^34^, and q^43^ (Figure 2) represent the transition intensities or hazards from underweight to healthy weight, healthy weight to underweight, healthy weight to overweight, overweight to healthy weight, overweight to obesity, and obesity to overweight, respectively. A zero in any row indicates that an instantaneous hazard is not allowed. The transition intensities in each row must sum to zero. Jackson provides further details on Markov multistate transition models [1].

3 Dataset extraction: primary analysis

The initial sample consisted of 18,294 children aged 9 months (Wave 1) when multiple births were excluded. Of these, 1,080 children with no records on the mother and implausible or missing bodyweight records were excluded. Of the remaining 17,214 children, 1,482 children were further excluded due to missing data on one or more covariates. The percentage missingness is as follows: ethnicity (0.15%), mother’s age at birth of child (0.01%), mother's BMI category during pregnancy (8.05%), mother's frequency of alcohol consumption during pregnancy (0.05%), mode of delivery (0.16%), mother's highest academic qualification (0.21%) and family income (0.3%). After excluding children with missing or implausible body weight records and/or missing relevant explanatory variables, the MCS cohort comprised 15,732 children aged 9 months (Wave 1).

Our samples and datesets were derived from the remaining 15,732 children if they had valid records on gestational age at birth and body weight recorded in subsequent Waves. Estimation of BMI z-scores using the Stata-based formulas for the British 1990 growth reference (UK90) [2] and the WHO growth reference [3] generated different missing values per dataset. We included children with valid z-scores based on both the UK90 and WHO references.

For the primary analysis, we extracted a dataset of children with measures of BMI for each wave (starting from Wave 2 when the children were three years old), which amounted to 11,731, 12,396, 11,325, 10,681, 9,126, and 7,768 children in Waves 2, 3, 4, 5, 6, and 7, respectively. We then extracted five pairwise datasets comprising sequential adjacent waves (i.e., Waves 2 and 3 (ages 3 and 5), 3 and 4 (ages 5 and 7), 4 and 5 (ages 7 and 11), waves 5 and 6 (ages 11 and 14), and 6 and 7 (ages 14 and 17)) with repeated BMI measurements as follows: 10,399 children for the Waves 2 to 3 dataset, 10,729 for the Waves 3 to 4 dataset, 9,685 children for the Waves 4 to 5 dataset, 8,593 children for the Waves 5 to 6 dataset, and 7,085 children for the Waves 6 to 7 dataset. Sociodemographic characteristics for waves 2 to 3 are reported in the main document. In this study, a pairwise dataset was constructed by matching the same child between adjacent or contiguous waves, where measures of BMI were available for both waves. Our piecewise linkage of the derived transition hazards between successive pairwise datasets denotes that transition hazard rates are assumed to be constant during the interval between two contiguous waves (within a pairwise dataset), but vary from one pairwise dataset to another [4-6].

# 4 Dataset extraction: scenario analyses

For scenario 1, children with a BMI record in Wave 2 and at least one BMI record in any subsequent wave were included in a single dataset, resulting in 42,047 observations or 7,502 children. Of these, 7,502, 7,452, 7,293, 6,812, and 5,486 observations represented the number of children with two, three, four, five, and six BMI records in successive waves, respectively. By default, the R MSM package for fitting multistate models to panel data will produce a transition intensity matrix for this dataset under the assumption of time homogeneity. The dataset for scenario 2 was the Waves 2 to 3 dataset described in the base case. Similarly, the R MSM package produces a single transition matrix for the scenario 2 dataset, which should fit any dataset constructed from contiguous waves or all the MCS waves if the assumption of time homogeneity holds. Our model validation exercise below investigates this assumption.

# 5 Observed prevalence

Table 1 Weighted prevalence based on the observed data from Waves 2 to 7 for 5,486 children with complete data on covariates and valid BMI z-scores using the UK90 reference and the British cut-offs for population monitoring

| Wave | Underweight | Healthy weight | Overweight | Obesity |
| --- | --- | --- | --- | --- |
| Wave 2 | 0.78% | 72.70% | 15.90% | 10.63% |
| Wave 3 | 0.59% | 76.59% | 13.86% | 8.96% |
| Wave 4 | 0.87% | 79.04% | 10.65% | 9.44% |
| Wave 5 | 1.40% | 68.21% | 14.79% | 15.60% |
| Wave 6 | 1.65% | 67.52% | 14.70% | 16.13% |
| Wave 7 | 1.69% | 66.54% | 14.12% | 17.66% |

Table 2 Unweighted prevalence based on the observed data from Waves 2 to 7 for 5,486 children with complete data on covariates and valid BMI z-scores using the UK90 reference and the British cut-offs for population monitoring

| Wave | Underweight | Healthy weight | Overweight | Obesity |
| --- | --- | --- | --- | --- |
| Wave 2 | 1.13% | 71.75% | 16.24% | 10.88% |
| Wave 3 | 0.98% | 76.01% | 13.60% | 9.41% |
| Wave 4 | 1.17% | 77.65% | 11.36% | 9.83% |
| Wave 5 | 1.60% | 66.88% | 14.86% | 16.66% |
| Wave 6 | 1.62% | 66.41% | 14.78% | 17.19% |
| Wave 7 | 1.93% | 65.09% | 14.27% | 18.70% |

Table 3 Weighted prevalence based on the observed data from Waves 2 to 7 for 5,486 children with complete data on covariates and valid BMI z-scores using the WHO references and cut-offs

| Wave | Underweight | Healthy weight | | Overweight | Obesity |
| --- | --- | --- | --- | --- | --- |
| Wave 2 | 0.31% | 88.23% | 9.52% | | 1.94% |
| Wave 3 | 0.35% | 71.07% | 22.16% | | 6.42% |
| Wave 4 | 0.68% | 75.18% | 17.47% | | 6.67% |
| Wave 5 | 1.44% | 66.34% | 22.11% | | 10.10% |
| Wave 6 | 2.02% | 70.61% | 19.21% | | 8.16% |
| Wave 7 | 1.93% | 70.64% | 18.32% | | 9.11% |

Table 4 Unweighted prevalence based on the observed data from Waves 2 to 7 for 5,486 children with complete data on covariates and valid BMI z-scores using the WHO references and cut-offs

| Wave | Underweight | Healthy weight | Overweight | Obesity |
| --- | --- | --- | --- | --- |
| Wave 2 | 0.51% | 87.81% | 9.53% | 2.15% |
| Wave 3 | 0.49% | 70.65% | 22.20% | 6.65% |
| Wave 4 | 0.89% | 74.04% | 17.86% | 7.20% |
| Wave 5 | 1.68% | 65.07% | 22.57% | 10.68% |
| Wave 6 | 1.97% | 69.60% | 19.98% | 8.46% |
| Wave 7 | 2.10% | 69.23% | 18.48% | 10.19% |

# 5 Model validation

Having derived transition intensities for our base case (primary analysis) and scenario models, we tested their performance in a validation dataset, which consisted of all children with complete and valid data on BMI measurements from wave 2 (age 3) through to wave 7 (age 17) of the Millennium Cohort Study (MCS). The number of children who met this criteria was 5,486. We adjusted the validation dataset by normalizing the wave 7 survey weights for each child to account for unequal sample selection probabilities and nonresponse. We multiplied each normalized weight by 100 and rounded to zero decimal places, such that children had weightings ranging from zero to 100. We then replicated each child to the number of times that corresponded to their normalized weightings. Finally, we combined the replicated dataset with the original validation dataset. We calculated the prevalence for each bodyweight category at baseline when children were 3 years old (Wave 2) and at subsequent waves in the new validation dataset comprising the original and replicated datasets. The baseline prevalence (Wave 2) of bodyweight categories from the validation dataset was entered as starting populations in Markov traces for our base case and scenario models, from which annual prevalence was estimated. To assess calibration or the degree of similarity between the observed data and estimates from the models, we compared the ratio of the modeled to the observed prevalence at each wave.

# 6 Incorporating complex survey design

The widely used R package for Markov multistate transition modelling [1] required modification to allow the incorporation of survey and replicate weights. We applied a validated adaptation that was used to analyze the Population Assessment of Tobacco and Health (PATH) study [7]. Both PATH and MCS provide survey weights that can be used to derive point estimates. However, a critical difference between the PATH study and our MCS analysis is that also PATH provides replicate weights, whereas MCS does not. When analyzing data from a complex sampling design, replicate weights are essential for variance estimation in the MSM framework.

Several replication methods are specified for variance estimation. Examples include the bootstrap method, balanced repeated replication (BRR), Fay’s method, and Jackknife methods (jk1, jk2, and jk-n). See <https://documentation.sas.com/doc/en/pgmsascdc/9.4_3.3/statug/statug_surveymeans_details50.htm> for further description of these methods. The jk-n is appropriate for sampling designs with two or more primary sampling units (PSUs) per stratum, such as the MCS, which has 9 strata and 398 PSUs. Other replication methods are inadequate for this study; for example, BRR is only suitable for designs with exactly two PSUs per stratum. Formulas and techniques for estimating replicate weights are well documented by Valliant and Dever [8].

# 7 Predicted-to-observed prevalence ratios

Table 5 British 1990 growth reference (UK90) and UK cut-offs for population monitoring: Ratio of predicted to observed prevalence in body weight status for the base case and scenario models (1 indicates complete agreement)

| **Age in years** | **Observed data** | **95% CI** | **Annual prevalence (base case)** | **95% CI** | **Ratio** | **Annual prevalence (scenario 1)** | **95% CI** | **Ratio** | **Annual prevalence (scenario 2)** | **95% CI** | **Ratio** |
| --- | --- | --- | --- | --- | --- | --- | --- | --- | --- | --- | --- |
|  |  |  |  |  | **(base case)** |  |  | **(scenario 1)** |  |  | **(scenario 2)** |
| **Underweight** |  |  |  |  |  |  |  |  |  |  |  |
| 3 | 0.0078 | 0.0074, 0.0081 | 0.0078 | 0.0074, 0.0081 | 1.0000 | 0.0078 | 0.0074, 0.0081 | 1.0000 | 0.0078 | 0.0074, 0.0081 | 1.0000 |
| 4 |  |  | 0.0074 | 0.0071, 0.0078 |  | 0.0080 | 0.0076, 0.0083 |  | 0.0074 | 0.0071, 0.0078 |  |
| 5 | 0.0059 | 0.0056, 0.0062 | 0.0072 | 0.0068, 0.0075 | 1.2207 | 0.0082 | 0.0078, 0.0085 | 1.3923 | 0.0072 | 0.0068, 0.0075 | 1.2207 |
| 6 |  |  | 0.0081 | 0.0078, 0.0085 |  | 0.0084 | 0.008, 0.0087 |  | 0.0070 | 0.0067, 0.0073 |  |
| 7 | 0.0087 | 0.0083, 0.0091 | 0.0088 | 0.0084, 0.0092 | 1.0143 | 0.0086 | 0.0082, 0.0089 | 0.9830 | 0.0069 | 0.0065, 0.0072 | 0.7918 |
| 8 |  |  | 0.0093 | 0.0089, 0.0097 |  | 0.0087 | 0.0084, 0.0091 |  | 0.0068 | 0.0065, 0.0072 |  |
| 9 |  |  | 0.0097 | 0.0093, 0.0101 |  | 0.0089 | 0.0085, 0.0093 |  | 0.0068 | 0.0064, 0.0071 |  |
| 10 |  |  | 0.0101 | 0.0096, 0.0105 |  | 0.0091 | 0.0087, 0.0095 |  | 0.0067 | 0.0064, 0.0071 |  |
| 11 | 0.0140 | 0.0135, 0.0145 | 0.0104 | 0.01, 0.0108 | 0.7442 | 0.0093 | 0.0089, 0.0097 | 0.6634 | 0.0067 | 0.0064, 0.0071 | 0.4821 |
| 12 |  |  | 0.0110 | 0.0106, 0.0114 |  | 0.0094 | 0.009, 0.0098 |  | 0.0067 | 0.0064, 0.0071 |  |
| 13 |  |  | 0.0115 | 0.0111, 0.012 |  | 0.0096 | 0.0092, 0.01 |  | 0.0067 | 0.0064, 0.0071 |  |
| 14 | 0.0165 | 0.0159, 0.017 | 0.0120 | 0.0116, 0.0125 | 0.7305 | 0.0098 | 0.0094, 0.0102 | 0.5927 | 0.0067 | 0.0064, 0.0071 | 0.4089 |
| 15 |  |  | 0.0127 | 0.0122, 0.0132 |  | 0.0099 | 0.0095, 0.0103 |  | 0.0067 | 0.0064, 0.0071 |  |
| 16 |  |  | 0.0133 | 0.0128, 0.0138 |  | 0.0101 | 0.0097, 0.0105 |  | 0.0067 | 0.0064, 0.0071 |  |
| 17 | 0.0169 | 0.0163, 0.0174 | 0.0138 | 0.0134, 0.0143 | 0.8200 | 0.0102 | 0.0098, 0.0106 | 0.6056 | 0.0068 | 0.0064, 0.0071 | 0.4000 |
| **Healthy weight** | |  |  |  |  |  |  |  |  |  |  |
| 3 | 0.7270 | 0.7251, 0.7288 | 0.7270 | 0.7251, 0.7288 | 1.0000 | 0.7270 | 0.7251, 0.7288 | 1.0000 | 0.7270 | 0.7251, 0.7288 | 1.0000 |
| 4 |  |  | 0.7343 | 0.7324, 0.7361 |  | 0.7266 | 0.7247, 0.7284 |  | 0.7343 | 0.7324, 0.7361 |  |
| 5 | 0.7659 | 0.7641, 0.7676 | 0.7400 | 0.7382, 0.7418 | 0.9662 | 0.7262 | 0.7244, 0.728 | 0.9482 | 0.7400 | 0.7382, 0.7418 | 0.9662 |
| 6 |  |  | 0.7448 | 0.743, 0.7466 |  | 0.7258 | 0.724, 0.7277 |  | 0.7446 | 0.7428, 0.7464 |  |
| 7 | 0.7904 | 0.7887, 0.7921 | 0.7478 | 0.746, 0.7496 | 0.9461 | 0.7254 | 0.7235, 0.7272 | 0.9178 | 0.7483 | 0.7465, 0.7501 | 0.9467 |
| 8 |  |  | 0.7394 | 0.7376, 0.7412 |  | 0.7250 | 0.7231, 0.7268 |  | 0.7514 | 0.7496, 0.7532 |  |
| 9 |  |  | 0.7313 | 0.7294, 0.7331 |  | 0.7246 | 0.7227, 0.7264 |  | 0.7541 | 0.7523, 0.7558 |  |
| 10 |  |  | 0.7234 | 0.7215, 0.7252 |  | 0.7241 | 0.7223, 0.726 |  | 0.7563 | 0.7545, 0.7581 |  |
| 11 | 0.6821 | 0.6802, 0.6841 | 0.7157 | 0.7138, 0.7175 | 1.0492 | 0.7237 | 0.7218, 0.7255 | 1.0609 | 0.7582 | 0.7565, 0.76 | 1.1116 |
| 12 |  |  | 0.7129 | 0.7111, 0.7148 |  | 0.7232 | 0.7214, 0.7251 |  | 0.7599 | 0.7582, 0.7617 |  |
| 13 |  |  | 0.7103 | 0.7085, 0.7122 |  | 0.7228 | 0.7209, 0.7246 |  | 0.7614 | 0.7596, 0.7632 |  |
| 14 | 0.6752 | 0.6732, 0.6771 | 0.7078 | 0.7059, 0.7097 | 1.0483 | 0.7223 | 0.7204, 0.7242 | 1.0698 | 0.7627 | 0.7609, 0.7644 | 1.1296 |
| 15 |  |  | 0.7049 | 0.703, 0.7068 |  | 0.7218 | 0.72, 0.7237 |  | 0.7638 | 0.7621, 0.7656 |  |
| 16 |  |  | 0.7019 | 0.7, 0.7038 |  | 0.7214 | 0.7195, 0.7232 |  | 0.7648 | 0.7631, 0.7666 |  |
| 17 | 0.6654 | 0.6634, 0.6673 | 0.6988 | 0.6969, 0.7007 | 1.0503 | 0.7209 | 0.719, 0.7227 | 1.0834 | 0.7657 | 0.764, 0.7675 | 1.1508 |
| **Overweight** | |  |  |  |  |  |  |  |  |  |  |
| 3 | 0.1590 | 0.1575, 0.1605 | 0.1590 | 0.1575, 0.1605 | 1.0000 | 0.1590 | 0.1575, 0.1605 | 1.0000 | 0.1590 | 0.1575, 0.1605 | 1.0000 |
| 4 |  |  | 0.1527 | 0.1512, 0.1541 |  | 0.1580 | 0.1565, 0.1596 |  | 0.1527 | 0.1512, 0.1541 |  |
| 5 | 0.1386 | 0.1372, 0.1401 | 0.1483 | 0.1468, 0.1498 | 1.0697 | 0.1571 | 0.1556, 0.1586 | 1.1336 | 0.1483 | 0.1468, 0.1498 | 1.0697 |
| 6 |  |  | 0.1369 | 0.1355, 0.1383 |  | 0.1563 | 0.1548, 0.1578 |  | 0.1452 | 0.1437, 0.1467 |  |
| 7 | 0.1065 | 0.1052, 0.1077 | 0.1292 | 0.1278, 0.1306 | 1.2138 | 0.1554 | 0.1539, 0.1569 | 1.4602 | 0.1430 | 0.1415, 0.1444 | 1.3429 |
| 8 |  |  | 0.1320 | 0.1306, 0.1334 |  | 0.1547 | 0.1532, 0.1561 |  | 0.1413 | 0.1398, 0.1427 |  |
| 9 |  |  | 0.1346 | 0.1332, 0.136 |  | 0.1539 | 0.1524, 0.1554 |  | 0.1400 | 0.1386, 0.1414 |  |
| 10 |  |  | 0.1369 | 0.1355, 0.1383 |  | 0.1532 | 0.1517, 0.1547 |  | 0.1390 | 0.1376, 0.1404 |  |
| 11 | 0.1479 | 0.1464, 0.1494 | 0.1390 | 0.1376, 0.1404 | 0.9397 | 0.1525 | 0.151, 0.154 | 1.0310 | 0.1382 | 0.1367, 0.1396 | 0.9342 |
| 12 |  |  | 0.1388 | 0.1374, 0.1403 |  | 0.1518 | 0.1503, 0.1533 |  | 0.1375 | 0.1361, 0.1389 |  |
| 13 |  |  | 0.1387 | 0.1373, 0.1402 |  | 0.1512 | 0.1497, 0.1527 |  | 0.1369 | 0.1355, 0.1384 |  |
| 14 | 0.1470 | 0.1455, 0.1485 | 0.1387 | 0.1373, 0.1401 | 0.9434 | 0.1506 | 0.1491, 0.1521 | 1.0244 | 0.1364 | 0.135, 0.1379 | 0.9281 |
| 15 |  |  | 0.1365 | 0.1351, 0.1379 |  | 0.1500 | 0.1486, 0.1515 |  | 0.1360 | 0.1346, 0.1374 |  |
| 16 |  |  | 0.1347 | 0.1333, 0.1361 |  | 0.1495 | 0.148, 0.151 |  | 0.1357 | 0.1342, 0.1371 |  |
| 17 | 0.1412 | 0.1397, 0.1426 | 0.1333 | 0.1319, 0.1347 | 0.9444 | 0.1490 | 0.1475, 0.1505 | 1.0552 | 0.1353 | 0.1339, 0.1368 | 0.9586 |
| **Obesity** |  |  |  |  |  |  |  |  |  |  |  |
| 3 | 0.1063 | 0.105, 0.1076 | 0.1063 | 0.105, 0.1076 | 1.0000 | 0.1063 | 0.105, 0.1076 | 1.0000 | 0.1063 | 0.105, 0.1076 | 1.0000 |
| 4 |  |  | 0.1057 | 0.1044, 0.1069 |  | 0.1074 | 0.1061, 0.1087 |  | 0.1057 | 0.1044, 0.1069 |  |
| 5 | 0.0896 | 0.0884, 0.0908 | 0.1046 | 0.1033, 0.1058 | 1.1665 | 0.1085 | 0.1072, 0.1098 | 1.2104 | 0.1046 | 0.1033, 0.1058 | 1.1665 |
| 6 |  |  | 0.1102 | 0.1089, 0.1115 |  | 0.1096 | 0.1083, 0.1109 |  | 0.1032 | 0.102, 0.1045 |  |
| 7 | 0.0944 | 0.0932, 0.0957 | 0.1142 | 0.1128, 0.1155 | 1.2087 | 0.1106 | 0.1093, 0.1119 | 1.1712 | 0.1018 | 0.1006, 0.1031 | 1.0784 |
| 8 |  |  | 0.1193 | 0.1179, 0.1206 |  | 0.1116 | 0.1103, 0.1129 |  | 0.1005 | 0.0992, 0.1017 |  |
| 9 |  |  | 0.1245 | 0.1231, 0.1258 |  | 0.1126 | 0.1113, 0.1139 |  | 0.0992 | 0.0979, 0.1004 |  |
| 10 |  |  | 0.1297 | 0.1283, 0.1311 |  | 0.1136 | 0.1123, 0.1149 |  | 0.0980 | 0.0967, 0.0992 |  |
| 11 | 0.1560 | 0.1545, 0.1575 | 0.1350 | 0.1335, 0.1364 | 0.8650 | 0.1146 | 0.1133, 0.1159 | 0.7344 | 0.0969 | 0.0956, 0.0981 | 0.6208 |
| 12 |  |  | 0.1372 | 0.1358, 0.1386 |  | 0.1155 | 0.1142, 0.1168 |  | 0.0959 | 0.0946, 0.0971 |  |
| 13 |  |  | 0.1394 | 0.138, 0.1408 |  | 0.1164 | 0.1151, 0.1178 |  | 0.0949 | 0.0937, 0.0962 |  |
| 14 | 0.1613 | 0.1598, 0.1629 | 0.1415 | 0.14, 0.1429 | 0.8768 | 0.1173 | 0.116, 0.1187 | 0.7272 | 0.0941 | 0.0929, 0.0953 | 0.5835 |
| 15 |  |  | 0.1460 | 0.1445, 0.1474 |  | 0.1182 | 0.1169, 0.1196 |  | 0.0934 | 0.0922, 0.0946 |  |
| 16 |  |  | 0.1501 | 0.1486, 0.1516 |  | 0.1191 | 0.1177, 0.1204 |  | 0.0928 | 0.0916, 0.094 |  |
| 17 | 0.1766 | 0.175, 0.1781 | 0.1540 | 0.1525, 0.1555 | 0.8722 | 0.1199 | 0.1186, 0.1213 | 0.6793 | 0.0922 | 0.091, 0.0934 | 0.5222 |

# 8 Sensitivity analysis


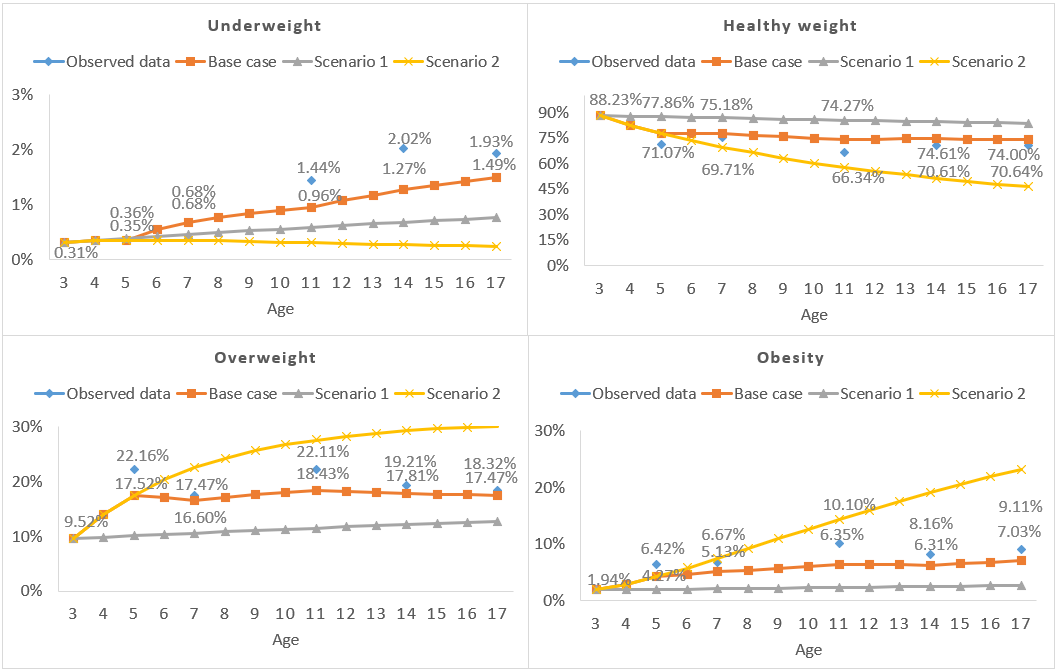


Figure 3 WHO references and cut-offs: Observed and modeled projections for survey-weighted children followed from age 3 to 17 years

Table 6 WHO references and cut-offs: Ratio of predicted to observed prevalence in body weight status for the base case and scenario models (1 indicates complete agreement)

| **Age in years** | **Observed data** | **95% CI** | **Annual prevalence (base case)** | **95% CI** | **Ratio** | **Annual prevalence (scenario 1)** | **95% CI** | **Ratio** | **Annual prevalence (scenario 2)** | **95% CI** | **Ratio** |
| --- | --- | --- | --- | --- | --- | --- | --- | --- | --- | --- | --- |
|  |  |  |  |  | **(base case)** |  |  | **(scenario 1)** |  |  | **(scenario 2)** |
| **Underweight** |  |  |  |  |  |  |  |  |  |  |  |
| 3 | 0.0031 | 0.0029, 0.0034 | 0.0031 | 0.0029, 0.0034 | 1.0000 | 0.0031 | 0.0029, 0.0034 | 1.0000 | 0.0031 | 0.0029, 0.0034 | 1.0000 |
| 4 |  |  | 0.0035 | 0.0032, 0.0037 |  | 0.0035 | 0.0033, 0.0038 |  | 0.0035 | 0.0032, 0.0037 |  |
| 5 | 0.0035 | 0.0032, 0.0037 | 0.0036 | 0.0033, 0.0038 | 1.0349 | 0.0039 | 0.0036, 0.0041 | 1.1166 | 0.0036 | 0.0033, 0.0038 | 1.0349 |
| 6 |  |  | 0.0055 | 0.0052, 0.0058 |  | 0.0042 | 0.004, 0.0045 |  | 0.0036 | 0.0034, 0.0039 |  |
| 7 | 0.0068 | 0.0065, 0.0072 | 0.0068 | 0.0065, 0.0072 | 1.0050 | 0.0046 | 0.0043, 0.0049 | 0.6742 | 0.0036 | 0.0033, 0.0038 | 0.5210 |
| 8 |  |  | 0.0076 | 0.0073, 0.008 |  | 0.0049 | 0.0046, 0.0052 |  | 0.0035 | 0.0032, 0.0037 |  |
| 9 |  |  | 0.0083 | 0.008, 0.0087 |  | 0.0053 | 0.005, 0.0056 |  | 0.0033 | 0.0031, 0.0036 |  |
| 10 |  |  | 0.0090 | 0.0086, 0.0094 |  | 0.0056 | 0.0053, 0.0059 |  | 0.0032 | 0.003, 0.0034 |  |
| 11 | 0.0144 | 0.0139, 0.0149 | 0.0096 | 0.0092, 0.01 | 0.6641 | 0.0059 | 0.0056, 0.0062 | 0.4110 | 0.0031 | 0.0029, 0.0033 | 0.2141 |
| 12 |  |  | 0.0107 | 0.0103, 0.0111 |  | 0.0062 | 0.0059, 0.0066 |  | 0.0030 | 0.0027, 0.0032 |  |
| 13 |  |  | 0.0118 | 0.0113, 0.0122 |  | 0.0065 | 0.0062, 0.0069 |  | 0.0029 | 0.0026, 0.0031 |  |
| 14 | 0.0202 | 0.0196, 0.0208 | 0.0127 | 0.0123, 0.0132 | 0.6303 | 0.0068 | 0.0065, 0.0072 | 0.3393 | 0.0027 | 0.0025, 0.003 | 0.1359 |
| 15 |  |  | 0.0135 | 0.013, 0.014 |  | 0.0071 | 0.0068, 0.0075 |  | 0.0026 | 0.0024, 0.0029 |  |
| 16 |  |  | 0.0143 | 0.0138, 0.0148 |  | 0.0074 | 0.0071, 0.0078 |  | 0.0025 | 0.0023, 0.0028 |  |
| 17 | 0.0193 | 0.0187, 0.0199 | 0.0149 | 0.0144, 0.0154 | 0.7724 | 0.0077 | 0.0073, 0.0081 | 0.3984 | 0.0025 | 0.0023, 0.0027 | 0.1273 |
| **Healthy weight** | |  |  |  |  |  |  |  |  |  |  |
| 3 | 0.8823 | 0.8809, 0.8836 | 0.8823 | 0.8809, 0.8836 | 1.0000 | 0.8823 | 0.8809, 0.8836 | 1.0000 | 0.8823 | 0.8809, 0.8836 | 1.0000 |
| 4 |  |  | 0.8271 | 0.8256, 0.8287 |  | 0.8787 | 0.8774, 0.8801 |  | 0.8271 | 0.8256, 0.8287 |  |
| 5 | 0.7107 | 0.7088, 0.7126 | 0.7786 | 0.7769, 0.7803 | 1.0956 | 0.8753 | 0.8739, 0.8766 | 1.2316 | 0.7786 | 0.7769, 0.7803 | 1.0956 |
| 6 |  |  | 0.7773 | 0.7755, 0.779 |  | 0.8718 | 0.8705, 0.8732 |  | 0.7355 | 0.7337, 0.7373 |  |
| 7 | 0.7518 | 0.75, 0.7536 | 0.7759 | 0.7741, 0.7776 | 1.0320 | 0.8685 | 0.8671, 0.8699 | 1.1552 | 0.6971 | 0.6952, 0.699 | 0.9272 |
| 8 |  |  | 0.7671 | 0.7654, 0.7689 |  | 0.8652 | 0.8638, 0.8666 |  | 0.6626 | 0.6607, 0.6646 |  |
| 9 |  |  | 0.7587 | 0.7569, 0.7605 |  | 0.8619 | 0.8605, 0.8634 |  | 0.6316 | 0.6296, 0.6336 |  |
| 10 |  |  | 0.7506 | 0.7488, 0.7523 |  | 0.8587 | 0.8573, 0.8602 |  | 0.6035 | 0.6015, 0.6055 |  |
| 11 | 0.6634 | 0.6615, 0.6654 | 0.7427 | 0.7409, 0.7445 | 1.1194 | 0.8556 | 0.8542, 0.8571 | 1.2897 | 0.5780 | 0.5759, 0.58 | 0.8712 |
| 12 |  |  | 0.7438 | 0.742, 0.7457 |  | 0.8525 | 0.851, 0.854 |  | 0.5547 | 0.5527, 0.5568 |  |
| 13 |  |  | 0.7450 | 0.7432, 0.7468 |  | 0.8495 | 0.848, 0.851 |  | 0.5335 | 0.5315, 0.5356 |  |
| 14 | 0.7061 | 0.7042, 0.708 | 0.7461 | 0.7443, 0.7479 | 1.0566 | 0.8465 | 0.845, 0.848 | 1.1989 | 0.5141 | 0.512, 0.5162 | 0.7281 |
| 15 |  |  | 0.7440 | 0.7422, 0.7458 |  | 0.8435 | 0.842, 0.845 |  | 0.4963 | 0.4942, 0.4984 |  |
| 16 |  |  | 0.7420 | 0.7402, 0.7438 |  | 0.8406 | 0.8391, 0.8422 |  | 0.4799 | 0.4778, 0.482 |  |
| 17 | 0.7064 | 0.7045, 0.7083 | 0.7400 | 0.7382, 0.7419 | 1.0476 | 0.8378 | 0.8363, 0.8393 | 1.1860 | 0.4648 | 0.4628, 0.4669 | 0.6580 |
| **Overweight** | |  |  |  |  |  |  |  |  |  |  |
| 3 | 0.0952 | 0.094, 0.0964 | 0.0952 | 0.094, 0.0964 | 1.0000 | 0.0952 | 0.094, 0.0964 | 1.0000 | 0.0952 | 0.094, 0.0964 | 1.0000 |
| 4 |  |  | 0.1399 | 0.1385, 0.1413 |  | 0.0979 | 0.0966, 0.0991 |  | 0.1399 | 0.1385, 0.1413 |  |
| 5 | 0.2216 | 0.2199, 0.2234 | 0.1752 | 0.1736, 0.1767 | 0.7904 | 0.1005 | 0.0992, 0.1017 | 0.4534 | 0.1752 | 0.1736, 0.1767 | 0.7904 |
| 6 |  |  | 0.1699 | 0.1684, 0.1715 |  | 0.1030 | 0.1018, 0.1043 |  | 0.2030 | 0.2013, 0.2046 |  |
| 7 | 0.1747 | 0.1731, 0.1763 | 0.1660 | 0.1645, 0.1676 | 0.9504 | 0.1055 | 0.1042, 0.1068 | 0.6040 | 0.2250 | 0.2232, 0.2267 | 1.2877 |
| 8 |  |  | 0.1710 | 0.1694, 0.1725 |  | 0.1079 | 0.1067, 0.1092 |  | 0.2423 | 0.2405, 0.2441 |  |
| 9 |  |  | 0.1757 | 0.1741, 0.1772 |  | 0.1103 | 0.109, 0.1116 |  | 0.2561 | 0.2543, 0.2579 |  |
| 10 |  |  | 0.1801 | 0.1785, 0.1817 |  | 0.1126 | 0.1113, 0.1139 |  | 0.2670 | 0.2651, 0.2688 |  |
| 11 | 0.2211 | 0.2194, 0.2229 | 0.1843 | 0.1826, 0.1859 | 0.8332 | 0.1149 | 0.1136, 0.1162 | 0.5195 | 0.2756 | 0.2738, 0.2775 | 1.2464 |
| 12 |  |  | 0.1820 | 0.1804, 0.1836 |  | 0.1171 | 0.1158, 0.1184 |  | 0.2825 | 0.2806, 0.2844 |  |
| 13 |  |  | 0.1800 | 0.1784, 0.1816 |  | 0.1193 | 0.1179, 0.1206 |  | 0.2880 | 0.2861, 0.2899 |  |
| 14 | 0.1921 | 0.1905, 0.1938 | 0.1781 | 0.1765, 0.1797 | 0.9269 | 0.1214 | 0.12, 0.1227 | 0.6316 | 0.2924 | 0.2905, 0.2942 | 1.5217 |
| 15 |  |  | 0.1768 | 0.1752, 0.1784 |  | 0.1234 | 0.122, 0.1248 |  | 0.2959 | 0.294, 0.2978 |  |
| 16 |  |  | 0.1757 | 0.1741, 0.1772 |  | 0.1254 | 0.124, 0.1268 |  | 0.2987 | 0.2968, 0.3006 |  |
| 17 | 0.1832 | 0.1816, 0.1848 | 0.1747 | 0.1731, 0.1763 | 0.9537 | 0.1274 | 0.126, 0.1288 | 0.6953 | 0.3009 | 0.299, 0.3028 | 1.6429 |
| **Obesity** |  |  |  |  |  |  |  |  |  |  |  |
| 3 | 0.0194 | 0.0188, 0.02 | 0.0194 | 0.0189, 0.02 | 1.0000 | 0.0194 | 0.0189, 0.02 | 1.0000 | 0.0194 | 0.0189, 0.02 | 1.0000 |
| 4 |  |  | 0.0295 | 0.0288, 0.0302 |  | 0.0199 | 0.0193, 0.0205 |  | 0.0295 | 0.0288, 0.0302 |  |
| 5 | 0.0642 | 0.0632, 0.0653 | 0.0427 | 0.0418, 0.0435 | 0.6641 | 0.0204 | 0.0198, 0.021 | 0.3173 | 0.0427 | 0.0418, 0.0435 | 0.6641 |
| 6 |  |  | 0.0473 | 0.0464, 0.0482 |  | 0.0209 | 0.0203, 0.0215 |  | 0.0579 | 0.0569, 0.0589 |  |
| 7 | 0.0667 | 0.0657, 0.0677 | 0.0513 | 0.0503, 0.0522 | 0.7684 | 0.0214 | 0.0208, 0.022 | 0.3208 | 0.0744 | 0.0733, 0.0755 | 1.1155 |
| 8 |  |  | 0.0542 | 0.0533, 0.0552 |  | 0.0219 | 0.0213, 0.0225 |  | 0.0916 | 0.0904, 0.0928 |  |
| 9 |  |  | 0.0573 | 0.0563, 0.0582 |  | 0.0225 | 0.0219, 0.0231 |  | 0.1090 | 0.1077, 0.1103 |  |
| 10 |  |  | 0.0604 | 0.0594, 0.0614 |  | 0.0230 | 0.0224, 0.0236 |  | 0.1263 | 0.125, 0.1277 |  |
| 11 | 0.1010 | 0.0998, 0.1023 | 0.0635 | 0.0625, 0.0645 | 0.6286 | 0.0236 | 0.0229, 0.0242 | 0.2334 | 0.1433 | 0.1419, 0.1448 | 1.4188 |
| 12 |  |  | 0.0634 | 0.0624, 0.0644 |  | 0.0241 | 0.0235, 0.0248 |  | 0.1598 | 0.1583, 0.1613 |  |
| 13 |  |  | 0.0633 | 0.0623, 0.0643 |  | 0.0247 | 0.0241, 0.0254 |  | 0.1756 | 0.1741, 0.1772 |  |
| 14 | 0.0816 | 0.0805, 0.0828 | 0.0631 | 0.0621, 0.0641 | 0.7736 | 0.0253 | 0.0247, 0.026 | 0.3101 | 0.1908 | 0.1892, 0.1924 | 2.3374 |
| 15 |  |  | 0.0657 | 0.0647, 0.0667 |  | 0.0259 | 0.0253, 0.0266 |  | 0.2052 | 0.2035, 0.2069 |  |
| 16 |  |  | 0.0681 | 0.067, 0.0691 |  | 0.0265 | 0.0259, 0.0272 |  | 0.2189 | 0.2171, 0.2206 |  |
| 17 | 0.0911 | 0.0899, 0.0923 | 0.0703 | 0.0693, 0.0714 | 0.7723 | 0.0271 | 0.0265, 0.0278 | 0.2979 | 0.2317 | 0.23, 0.2335 | 2.5445 |

Table 7 Sensitivity analysis: unadjusted estimates of transition hazards derived before excluding children with incomplete data on covariates

| **To From** | **Underweight** |  | **Healthy weight** |  | **Overweight** |  | **Obesity** |  |  |
| --- | --- | --- | --- | --- | --- | --- | --- | --- | --- |
|  |  |  |  |  |  |  |  |  |  |
|  | **MLE** | **95% CI** | **MLE** | **95% CI** | **MLE** | **95% CI** | **MLE** | **95% CI** |  |
| **Waves 2 (mean age 3) to 3 (mean age 5), n = 11,248** | | | | | | | | |  |
| **Underweight** | -0.6323 | -0.7893, -0.5066 | 0.6323 | 0.5066, 0.7893 | 0 | 0, 0 | 0 | 0, 0 |  |
| **Healthy weight** | 0.0064 | 0.0048, 0.0084 | -0.0869 | -0.0952, -0.0796 | 0.0806 | 0.0748, 0.0868 | 0 | 0, 0 |  |
| **Overweight** | 0 | 0, 0 | 0.4669 | 0.4369, 0.4989 | -0.714 | -0.7739, -0.6589 | 0.2471 | 0.222, 0.275 |  |
| **Obesity** | 0 | 0, 0 | 0 | 0, 0 | 0.3669 | 0.3341, 0.4028 | -0.3669 | -0.4028, -0.3341 |  |
| **Waves 3 (mean age 5) to 4 (mean age 7), n = 11,629** | | | | | | | | |  |
| **Underweight** | -0.6308 | -0.827, -0.4812 | 0.6308 | 0.4812, 0.827 | 0 | 0, 0 | 0 | 0, 0 |  |
| **Healthy weight** | 0.0093 | 0.0072, 0.0119 | -0.0737 | -0.0816, -0.0668 | 0.0645 | 0.0596, 0.0697 | 0 | 0, 0 |  |
| **Overweight** | 0 | 0, 0 | 0.4125 | 0.3831, 0.4441 | -0.6558 | -0.7128, -0.6034 | 0.2433 | 0.2203, 0.2687 |  |
| **Obesity** | 0 | 0, 0 | 0 | 0, 0 | 0.2116 | 0.1892, 0.2366 | -0.2116 | -0.2366, -0.1892 |  |
| **Waves 4 (mean age 7) to 5 (mean age 11), n = 10,490** | | | | | | | | |  |
| **Underweight** | -0.3013 | -0.3864, -0.2349 | 0.3013 | 0.2349, 0.3864 | 0 | 0, 0 | 0 | 0, 0 |  |
| **Healthy weight** | 0.0061 | 0.0048, 0.0077 | -0.0698 | -0.0753, -0.065 | 0.0637 | 0.0602, 0.0675 | 0 | 0, 0 |  |
| **Overweight** | 0 | 0, 0 | 0.1086 | 0.096, 0.1229 | -0.3266 | -0.3581, -0.298 | 0.2179 | 0.2019, 0.2352 |  |
| **Obesity** | 0 | 0, 0 | 0 | 0, 0 | 0.0675 | 0.0578, 0.0787 | -0.0675 | -0.0787, -0.0578 |  |
| **Waves 5 (mean age 11) to 6 (mean age 14), n = 9,322** | | | | | | | | |  |
| **Underweight** | -0.3107 | -0.3911, -0.2468 | 0.3107 | 0.2468, 0.3911 | 0 | 0, 0 | 0 | 0, 0 |  |
| **Healthy weight** | 0.0075 | 0.0059, 0.0095 | -0.0634 | -0.0702, -0.0574 | 0.0559 | 0.0515, 0.0607 | 0 | 0, 0 |  |
| **Overweight** | 0 | 0, 0 | 0.2464 | 0.2267, 0.2679 | -0.4313 | -0.4719, -0.3943 | 0.1849 | 0.1676, 0.204 |  |
| **Obesity** | 0 | 0, 0 | 0 | 0, 0 | 0.1229 | 0.111, 0.1361 | -0.1229 | -0.1361, -0.111 |  |
| **Waves 6 (mean age 14) to 7 (mean age 17) n = 7,701** | | | | | | | | |  |
| **Underweight** | -0.2532 | -0.3345, -0.1917 | 0.2532 | 0.1917, 0.3345 | 0 | 0, 0 | 0 | 0, 0 |  |
| **Healthy weight** | 0.0071 | 0.0054, 0.0094 | -0.0677 | -0.0761, -0.0604 | 0.0606 | 0.055, 0.0668 | 0 | 0, 0 |  |
| **Overweight** | 0 | 0, 0 | 0.2639 | 0.2378, 0.2929 | -0.493 | -0.5496, -0.4422 | 0.229 | 0.2044, 0.2567 |  |
| **Obesity** | 0 | 0, 0 | 0 | 0, 0 | 0.1267 | 0.1109, 0.1447 | -0.1267 | -0.1447, -0.1109 |  |

MLE: Maximum likelihood estimate of transition hazard. Point estimates incorporate survey weights. British 1990 growth reference (UK90) and UK cut-offs for population monitoring

Table 8 Primary analysis: unadjusted estimates of transition hazards derived after excluding children with incomplete data on covariates

| **To From** | **Underweight** |  | **Healthy weight** |  | **Overweight** |  | **Obesity** |  |  |
| --- | --- | --- | --- | --- | --- | --- | --- | --- | --- |
|  |  |  |  |  |  |  |  |  |  |
|  | **MLE** | **95% CI** | **MLE** | **95% CI** | **MLE** | **95% CI** | **MLE** | **95% CI** |  |
| **Waves 2 (mean age 3) to 3 (mean age 5), n = 10,399** | | | | | | | | |  |
| **Underweight** | -0.6503 | -1.5159, -0.279 | 0.6503 | 0.279, 1.5159 | 0 | 0, 0 | 0 | 0, 0 |  |
| **Healthy weight** | 0.0057 | 0.0022, 0.0154 | -0.0859 | -0.1225, -0.0622 | 0.0802 | 0.06, 0.1071 | 0 | 0, 0 |  |
| **Overweight** | 0 | 0, 0 | 0.4661 | 0.3529, 0.6157 | -0.7101 | -0.9801, -0.5162 | 0.244 | 0.1633, 0.3644 |  |
| **Obesity** | 0 | 0, 0 | 0 | 0, 0 | 0.3694 | 0.2613, 0.5221 | -0.3694 | -0.5221, -0.2613 |  |
| **Waves 3 (mean age 5) to 4 (mean age 7), n = 10,729** | | | | | | | | |  |
| **Underweight** | -0.652 | -1.8784, -0.2263 | 0.652 | 0.2263, 1.8784 | 0 | 0, 0 | 0 | 0, 0 |  |
| **Healthy weight** | 0.0093 | 0.0038, 0.0229 | -0.073 | -0.1068, -0.0521 | 0.0637 | 0.0483, 0.0839 | 0 | 0, 0 |  |
| **Overweight** | 0 | 0, 0 | 0.4138 | 0.3171, 0.5399 | -0.6559 | -0.892, -0.4836 | 0.2421 | 0.1665, 0.3521 |  |
| **Obesity** | 0 | 0, 0 | 0 | 0, 0 | 0.2164 | 0.1437, 0.3258 | -0.2164 | -0.3258, -0.1437 |  |
| **Waves 4 (mean age 7) to 5 (mean age 11), n = 9,685** | | | | | | | | |  |
| **Underweight** | -0.2795 | -0.7281, -0.1073 | 0.2795 | 0.1073, 0.7281 | 0 | 0, 0 | 0 | 0, 0 |  |
| **Healthy weight** | 0.0059 | 0.0026, 0.0135 | -0.0673 | -0.0898, -0.052 | 0.0614 | 0.0494, 0.0763 | 0 | 0, 0 |  |
| **Overweight** | 0 | 0, 0 | 0.1062 | 0.0645, 0.1749 | -0.3265 | -0.4677, -0.2302 | 0.2203 | 0.1657, 0.2928 |  |
| **Obesity** | 0 | 0, 0 | 0 | 0, 0 | 0.0705 | 0.0402, 0.1234 | -0.0705 | -0.1234, -0.0402 |  |
| **Waves 5 (mean age 11) to 6 (mean age 14), n = 8,593** | | | | | | | | |  |
| **Underweight** | -0.3024 | -0.8045, -0.1137 | 0.3024 | 0.1137, 0.8045 | 0 | 0, 0 | 0 | 0, 0 |  |
| **Healthy weight** | 0.0071 | 0.0029, 0.0178 | -0.0638 | -0.0948, -0.0447 | 0.0567 | 0.0418, 0.077 | 0 | 0, 0 |  |
| **Overweight** | 0 | 0, 0 | 0.2462 | 0.1742, 0.348 | -0.4182 | -0.6143, -0.2853 | 0.172 | 0.1111, 0.2663 |  |
| **Obesity** | 0 | 0, 0 | 0 | 0, 0 | 0.1253 | 0.0825, 0.1902 | -0.1253 | -0.1902, -0.0825 |  |
| **Waves 6 (mean age 14) to 7 (mean age 17) n = 7,085** | | | | | | | | |  |
| **Underweight** | -0.2549 | -0.6035, -0.1077 | 0.2549 | 0.1077, 0.6035 | 0 | 0, 0 | 0 | 0, 0 |  |
| **Healthy weight** | 0.0073 | 0.002, 0.0267 | -0.0675 | -0.1134, -0.0438 | 0.0602 | 0.0418, 0.0867 | 0 | 0, 0 |  |
| **Overweight** | 0 | 0, 0 | 0.259 | 0.1749, 0.3834 | -0.4931 | -0.8057, -0.3047 | 0.2341 | 0.1298, 0.4223 |  |
| **Obesity** | 0 | 0, 0 | 0 | 0, 0 | 0.13 | 0.0782, 0.2161 | -0.13 | -0.2161, -0.0782 |  |

MLE: Maximum likelihood estimate of transition hazard. Point estimates incorporate survey weights, and confidence intervals were derived using replicate weights. British 1990 growth reference (UK90) and UK cut-offs for population monitoring.

Table 9 WHO references and cut-offs: unadjusted estimates of annual transition probabilities for transitions between weight status categories

| **To From** | **Underweight** |  | **Healthy weight** |  | **Overweight** |  | **Obesity** |  |  |
| --- | --- | --- | --- | --- | --- | --- | --- | --- | --- |
|  |  |  |  |  |  |  |  |  |  |
|  | **Annual probability** | **95% CI** | **Annual probability** | **95% CI** | **Annual probability** | **95% CI** | **Annual probability** | **95% CI** |  |
| **Waves 2 (mean age 3) to 3 (mean age 5), n = 10,399** | | | | | | | | |  |
| **Underweight** | 0.6523 | 0.1805, 0.8995 | 0.3347 | 0.0974, 0.7776 | 0.0126 | 0.003, 0.0399 | 0.0004 | 0.0001, 0.002 |  |
| **Healthy weight** | 0.0016 | 0.0004, 0.0042 | 0.9301 | 0.9178, 0.9405 | 0.0650 | 0.057, 0.0731 | 0.0033 | 0.0021, 0.0049 |  |
| **Overweight** | 0.0001 | 0, 0.0003 | 0.0574 | 0.0337, 0.0961 | 0.8530 | 0.7935, 0.8969 | 0.0895 | 0.0694, 0.1101 |  |
| **Obesity** | 0.0000 | 0, 0 | 0.0022 | 0.0005, 0.0092 | 0.0683 | 0.0285, 0.1562 | 0.9295 | 0.8346, 0.971 |  |
| **Waves 3 (mean age 5) to 4 (mean age 7), n = 10,729** | | | | | | | | |  |
| **Underweight** | 0.7171 | 0.2362, 0.9268 | 0.2784 | 0.0724, 0.7456 | 0.0044 | 0.0008, 0.0177 | 0.0001 | 0, 0.0005 |  |
| **Healthy weight** | 0.0037 | 0.0015, 0.0064 | 0.9674 | 0.9563, 0.9763 | 0.0281 | 0.0218, 0.0359 | 0.0008 | 0.0004, 0.0014 |  |
| **Overweight** | 0.0002 | 0, 0.0006 | 0.1300 | 0.1075, 0.1559 | 0.8233 | 0.7791, 0.8595 | 0.0465 | 0.033, 0.0644 |  |
| **Obesity** | 0.0000 | 0, 0 | 0.0068 | 0.0036, 0.0125 | 0.0898 | 0.0607, 0.1307 | 0.9034 | 0.8568, 0.9357 |  |
| **Waves 4 (mean age 7) to 5 (mean age 11), n = 9,685** | | | | | | | | |  |
| **Underweight** | 0.9323 | 0.8044, 0.9777 | 0.0672 | 0.0222, 0.1938 | 0.0005 | 0.0001, 0.0018 | 0.0000 | 0, 0 |  |
| **Healthy weight** | 0.0016 | 0.0007, 0.0036 | 0.9838 | 0.9783, 0.9875 | 0.0144 | 0.0117, 0.0178 | 0.0002 | 0.0001, 0.0003 |  |
| **Overweight** | 0.0000 | 0, 0.0001 | 0.0203 | 0.0134, 0.0309 | 0.9566 | 0.9383, 0.9694 | 0.0231 | 0.0172, 0.0307 |  |
| **Obesity** | 0.0000 | 0, 0 | 0.0002 | 0.0001, 0.0005 | 0.0194 | 0.0115, 0.0325 | 0.9804 | 0.967, 0.9884 |  |
| **Waves 5 (mean age 11) to 6 (mean age 14), n = 8,593** | | | | | | | | |  |
| **Underweight** | 0.9151 | 0.7964, 0.9661 | 0.0844 | 0.0338, 0.2018 | 0.0005 | 0.0001, 0.0018 | 0.0000 | 0, 0 |  |
| **Healthy weight** | 0.0026 | 0.0011, 0.0057 | 0.9856 | 0.9777, 0.9905 | 0.0117 | 0.0084, 0.0163 | 0.0001 | 0, 0.0003 |  |
| **Overweight** | 0.0001 | 0, 0.0002 | 0.0595 | 0.0457, 0.0768 | 0.9220 | 0.8914, 0.9437 | 0.0184 | 0.0106, 0.0316 |  |
| **Obesity** | 0.0000 | 0, 0 | 0.0017 | 0.0009, 0.0033 | 0.0542 | 0.0365, 0.0796 | 0.9441 | 0.9171, 0.9626 |  |
| **Waves 6 (mean age 14) to 7 (mean age 17) n = 7,085** | | | | | | | | |  |
| **Underweight** | 0.9086 | 0.8002, 0.9598 | 0.0907 | 0.04, 0.1977 | 0.0007 | 0.0002, 0.0021 | 0.0000 | 0, 0 |  |
| **Healthy weight** | 0.0026 | 0.0008, 0.0079 | 0.9832 | 0.9715, 0.9894 | 0.0140 | 0.0097, 0.0201 | 0.0002 | 0.0001, 0.0005 |  |
| **Overweight** | 0.0002 | 0, 0.0003 | 0.0520 | 0.035, 0.0764 | 0.9196 | 0.8762, 0.9483 | 0.0282 | 0.0167, 0.0471 |  |
| **Obesity** | 0.0000 | 0, 0 | 0.0011 | 0.0004, 0.003 | 0.0406 | 0.0233, 0.0695 | 0.9583 | 0.9275, 0.9763 |  |

Point estimates incorporate survey weights, and confidence intervals were derived using replicate weights


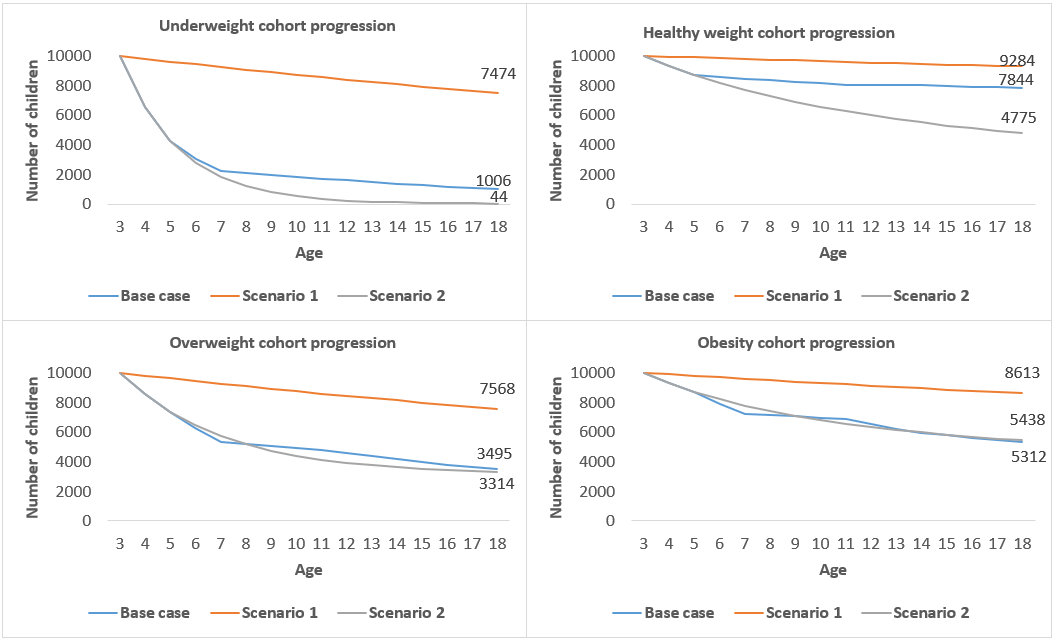


Figure 4 WHO references and cut-offs: Modeled prevalence for four hypothetical cohorts of 10,000 children aged three at baseline in underweight, healthy weight, overweight, and obesity categories

# 9 Hazard ratios for explanatory variables

Table 10 Primary analysis: hazard ratios of transitions between weight status categories for explanatory variables for Waves 2-3 (ages 3-5 years)

| **Transition** | **Estimate** | **Lower 95% CI** | **Upper CI 95% CI** |
| --- | --- | --- | --- |
| **Ethnicity: None-white versus White** | | | |
| Underweight to Healthy weight | 0.3678 | 0.1533 | 0.8829 |
| Healthy weight to Underweight | 2.1510 | 1.4377 | 3.2182 |
| Healthy weight to Overweight | 1.1127 | 0.6327 | 1.9571 |
| Overweight to Healthy weight | 1.0034 | 0.5947 | 1.6931 |
| Overweight to Obesity | 1.3304 | 0.6641 | 2.6649 |
| Obesity to Overweight | 0.7917 | 0.3921 | 1.5986 |
| **Gestational age at birth: Early term versus Preterm** | | | |
| Underweight to Healthy weight | 1.3012 | 1.2621 | 1.3415 |
| Healthy weight to Underweight | 0.4104 | 0.3867 | 0.4357 |
| Healthy weight to Overweight | 0.7493 | 0.5006 | 1.1216 |
| Overweight to Healthy weight | 1.1271 | 0.6516 | 1.9498 |
| Overweight to Obesity | 1.4264 | 0.8831 | 2.3041 |
| Obesity to Overweight | 1.4968 | 0.7649 | 2.9290 |
| **Gestational age at birth: Full term versus Preterm** | | | |
| Underweight to Healthy weight | 1.7352 | 0.6782 | 4.4396 |
| Healthy weight to Underweight | 0.6197 | 0.2277 | 1.6868 |
| Healthy weight to Overweight | 0.8207 | 0.4341 | 1.5515 |
| Overweight to Healthy weight | 1.0325 | 0.4632 | 2.3014 |
| Overweight to Obesity | 1.1938 | 0.4743 | 3.0051 |
| Obesity to Overweight | 1.6642 | 0.6030 | 4.5929 |
| **Gestational age at birth: Late term versus Preterm** | | | |
| Underweight to Healthy weight | 1.4176 | 1.3062 | 1.5385 |
| Healthy weight to Underweight | 0.2412 | 0.1522 | 0.3822 |
| Healthy weight to Overweight | 0.8376 | 0.5822 | 1.2051 |
| Overweight to Healthy weight | 0.9424 | 0.5316 | 1.6707 |
| Overweight to Obesity | 1.2386 | 0.6923 | 2.2158 |
| Obesity to Overweight | 1.6260 | 0.7580 | 3.4876 |
| **Gestational age at birth: Postterm versus Preterm** | | | |
| Underweight to Healthy weight | 1.8279 | 0.0989 | 33.7671 |
| Healthy weight to Underweight | 1.5103 | 0.1823 | 12.5135 |
| Healthy weight to Overweight | 0.7435 | 0.4995 | 1.1066 |
| Overweight to Healthy weight | 0.8952 | 0.8632 | 0.9285 |
| Overweight to Obesity | 1.1118 | 0.7662 | 1.6133 |
| Obesity to Overweight | 0.9708 | 0.6411 | 1.4700 |
| **Mother's age at birth of child: 20 to 29 versus 12 to 19** | | | |
| Underweight to Healthy weight | 0.2347 | 0.0277 | 1.9908 |
| Healthy weight to Underweight | 0.2119 | 0.0102 | 4.3876 |
| Healthy weight to Overweight | 1.0065 | 0.5642 | 1.7955 |
| Overweight to Healthy weight | 1.2527 | 0.6707 | 2.3397 |
| Overweight to Obesity | 1.5805 | 0.4833 | 5.1679 |
| Obesity to Overweight | 1.1069 | 0.4335 | 2.8265 |
| **Mother's age at birth of child: 30 and over versus 12 to 19** | | | |
| Underweight to Healthy weight | 0.2564 | 0.0374 | 1.7565 |
| Healthy weight to Underweight | 0.4179 | 0.0197 | 8.8644 |
| Healthy weight to Overweight | 0.9557 | 0.5305 | 1.7216 |
| Overweight to Healthy weight | 1.2348 | 0.6439 | 2.3681 |
| Overweight to Obesity | 1.2164 | 0.3421 | 4.3254 |
| Obesity to Overweight | 0.8831 | 0.3338 | 2.3364 |
| **Mother's BMI category during pregnancy: Underweight versus Healthy weight** | | | |
| Underweight to Healthy weight | 0.5495 | 0.0233 | 12.9557 |
| Healthy weight to Underweight | 3.4408 | 1.0325 | 11.4670 |
| Healthy weight to Overweight | 0.4988 | 0.1512 | 1.6454 |
| Overweight to Healthy weight | 0.8862 | 0.3070 | 2.5582 |
| Overweight to Obesity | 1.3547 | 0.2484 | 7.3871 |
| Obesity to Overweight | 1.1655 | 0.2467 | 5.5064 |
| **Mother's BMI category during pregnancy: Overweight/obesity versus Healthy weight** | | | |
| Underweight to Healthy weight | 1.8999 | 0.6911 | 5.2231 |
| Healthy weight to Underweight | 1.5651 | 0.3678 | 6.6595 |
| Healthy weight to Overweight | 1.5604 | 1.3981 | 1.7416 |
| Overweight to Healthy weight | 0.7090 | 0.6265 | 0.8024 |
| Overweight to Obesity | 1.1315 | 1.1068 | 1.1569 |
| Obesity to Overweight | 0.6292 | 0.5978 | 0.6621 |
| **Mother's frequency of alcohol consumption during pregnancy: Monthly or more frequently versus Never** | | | |
| Underweight to Healthy weight | 1.0696 | 0.0006 | 1974.7089 |
| Healthy weight to Underweight | 0.3341 | 0.0006 | 194.2117 |
| Healthy weight to Overweight | 0.7443 | 0.5031 | 1.1009 |
| Overweight to Healthy weight | 0.9696 | 0.7577 | 1.2407 |
| Overweight to Obesity | 0.9519 | 0.6496 | 1.3950 |
| Obesity to Overweight | 1.0401 | 0.6714 | 1.6114 |
| **Mother's frequency of alcohol consumption during pregnancy: Less than monthly versus Never** | | | |
| Underweight to Healthy weight | 0.9510 | 0.1290 | 7.0130 |
| Healthy weight to Underweight | 1.5395 | 0.3604 | 6.5765 |
| Healthy weight to Overweight | 0.8337 | 0.5696 | 1.2202 |
| Overweight to Healthy weight | 0.9123 | 0.6610 | 1.2590 |
| Overweight to Obesity | 1.3815 | 0.8251 | 2.3132 |
| Obesity to Overweight | 1.4954 | 0.9672 | 2.3120 |
| **Mode of delivery: Assisted versus Normal** | | | |
| Underweight to Healthy weight | 1.6352 | 0.5455 | 4.9021 |
| Healthy weight to Underweight | 2.0876 | 0.1205 | 36.1722 |
| Healthy weight to Overweight | 1.0239 | 0.6814 | 1.5386 |
| Overweight to Healthy weight | 0.8241 | 0.5282 | 1.2858 |
| Overweight to Obesity | 0.7033 | 0.2722 | 1.8172 |
| Obesity to Overweight | 0.8927 | 0.4722 | 1.6876 |
| **Mode of delivery: Planned C-section versus Normal** | | | |
| Underweight to Healthy weight | 1.3663 | 0.0146 | 127.9112 |
| Healthy weight to Underweight | 3.3230 | 0.0486 | 227.2999 |
| Healthy weight to Overweight | 0.8705 | 0.4688 | 1.6164 |
| Overweight to Healthy weight | 0.9126 | 0.5294 | 1.5733 |
| Overweight to Obesity | 0.9142 | 0.4621 | 1.8088 |
| Obesity to Overweight | 0.7139 | 0.3238 | 1.5742 |
| **Mode of delivery: Emergency C-section versus Normal** | | | |
| Underweight to Healthy weight | 0.7150 | 0.2818 | 1.8139 |
| Healthy weight to Underweight | 1.5273 | 0.3489 | 6.6851 |
| Healthy weight to Overweight | 0.9529 | 0.6361 | 1.4276 |
| Overweight to Healthy weight | 0.7280 | 0.4894 | 1.0828 |
| Overweight to Obesity | 1.2291 | 0.6097 | 2.4777 |
| Obesity to Overweight | 1.1922 | 0.6486 | 2.1911 |
| **Mother's highest academic qualification: Diplomas versus Degree** | | | |
| Underweight to Healthy weight | 1.3337 | 0.1086 | 16.3778 |
| Healthy weight to Underweight | 0.5733 | 0.0738 | 4.4525 |
| Healthy weight to Overweight | 1.1242 | 0.8690 | 1.4544 |
| Overweight to Healthy weight | 0.9410 | 0.7659 | 1.1561 |
| Overweight to Obesity | 1.3402 | 0.9862 | 1.8214 |
| Obesity to Overweight | 0.9572 | 0.6748 | 1.3578 |
| **Mother's highest academic qualification: A levels versus degree** | | | |
| Underweight to Healthy weight | 2.1883 | 0.2986 | 16.0394 |
| Healthy weight to Underweight | 0.7443 | 0.0896 | 6.1829 |
| Healthy weight to Overweight | 1.4411 | 1.2651 | 1.6415 |
| Overweight to Healthy weight | 1.0566 | 0.8801 | 1.2686 |
| Overweight to Obesity | 0.8852 | 0.6295 | 1.2448 |
| Obesity to Overweight | 1.0547 | 0.8494 | 1.3095 |
| **Mother's highest academic qualification: O levels versus degree** | | | |
| Underweight to Healthy weight | 1.3986 | 1.1922 | 1.6407 |
| Healthy weight to Underweight | 1.4939 | 0.4538 | 4.9178 |
| Healthy weight to Overweight | 1.5239 | 1.1415 | 2.0344 |
| Overweight to Healthy weight | 1.0015 | 0.9422 | 1.0645 |
| Overweight to Obesity | 1.2628 | 0.9176 | 1.7380 |
| Obesity to Overweight | 0.8610 | 0.6829 | 1.0855 |
| **Mother's highest academic qualification: GCSE D G versus Degree** | | | |
| Underweight to Healthy weight | 1.5850 | 0.1913 | 13.1352 |
| Healthy weight to Underweight | 1.2865 | 0.7821 | 2.1161 |
| Healthy weight to Overweight | 1.5135 | 1.3820 | 1.6575 |
| Overweight to Healthy weight | 0.8863 | 0.6654 | 1.1805 |
| Overweight to Obesity | 1.1914 | 1.0045 | 1.4131 |
| Obesity to Overweight | 0.7937 | 0.6414 | 0.9821 |
| **Mother's highest academic qualification: Other versus Degree** | | | |
| Underweight to Healthy weight | 2.8722 | 0.1588 | 51.9360 |
| Healthy weight to Underweight | 5.8279 | 0.4293 | 79.1193 |
| Healthy weight to Overweight | 1.5500 | 0.5184 | 4.6346 |
| Overweight to Healthy weight | 0.7921 | 0.1887 | 3.3247 |
| Overweight to Obesity | 1.6253 | 0.3949 | 6.6889 |
| Obesity to Overweight | 0.5913 | 0.0654 | 5.3473 |
| **Mother's highest academic qualification: None versus Degree** | | | |
| Underweight to Healthy weight | 1.2929 | 1.1639 | 1.4363 |
| Healthy weight to Underweight | 1.9423 | 1.0077 | 3.7439 |
| Healthy weight to Overweight | 1.8226 | 1.7165 | 1.9351 |
| Overweight to Healthy weight | 1.0523 | 0.8884 | 1.2464 |
| Overweight to Obesity | 2.2404 | 1.5479 | 3.2429 |
| Obesity to Overweight | 1.3421 | 1.0609 | 1.6977 |
| **Family income: Lowest quintile versus Highest quintile** | | | |
| Underweight to Healthy weight | 1.3331 | 1.2548 | 1.4163 |
| Healthy weight to Underweight | 1.7105 | 1.1677 | 2.5057 |
| Healthy weight to Overweight | 1.5544 | 1.4516 | 1.6646 |
| Overweight to Healthy weight | 0.8815 | 0.7908 | 0.9825 |
| Overweight to Obesity | 1.3520 | 1.3452 | 1.3589 |
| Obesity to Overweight | 0.8792 | 0.7081 | 1.0915 |
| **Family income: Second quintile versus Highest quintile** | | | |
| Underweight to Healthy weight | 1.5798 | 0.6050 | 4.1254 |
| Healthy weight to Underweight | 2.0106 | 1.2304 | 3.2857 |
| Healthy weight to Overweight | 1.1117 | 1.0611 | 1.1648 |
| Overweight to Healthy weight | 0.8096 | 0.7649 | 0.8568 |
| Overweight to Obesity | 1.0154 | 0.9183 | 1.1229 |
| Obesity to Overweight | 0.9531 | 0.9383 | 0.9681 |
| **Family income: Third quintile versus Highest quintile** | | | |
| Underweight to Healthy weight | 0.9645 | 0.8619 | 1.0792 |
| Healthy weight to Underweight | 1.3398 | 1.0247 | 1.7518 |
| Healthy weight to Overweight | 1.3970 | 1.3622 | 1.4328 |
| Overweight to Healthy weight | 0.8311 | 0.8200 | 0.8423 |
| Overweight to Obesity | 0.9554 | 0.9428 | 0.9681 |
| Obesity to Overweight | 0.9537 | 0.9351 | 0.9726 |
| **Family income: Fourth quintile versus Highest quintile** | | | |
| Underweight to Healthy weight | 2.0835 | 1.8819 | 2.3066 |
| Healthy weight to Underweight | 2.5822 | 2.3607 | 2.8246 |
| Healthy weight to Overweight | 1.1440 | 1.1324 | 1.1558 |
| Overweight to Healthy weight | 0.7755 | 0.7670 | 0.7842 |
| Overweight to Obesity | 0.9795 | 0.8768 | 1.0941 |
| Obesity to Overweight | 0.8167 | 0.7539 | 0.8848 |

# 10 Checking for model convergence

When estimating transition hazards, the model may not converge if there are no observed transitions within the data or if the number of transitions from one state to another is too small. This non-convergence and, ultimately, failure of the model to produce a maximum likelihood estimate is often due to the small sample size of the affected subgroup within an explanatory variable. Lack of convergence may not be evident from merely observing transition hazards. Therefore, it is necessary to calculate transition probabilities and their confidence intervals. If convergence is not achieved, the transition probabilities on a transition matrix row will not sum up to 1 (usually, they will exceed 1). A fix for non-convergence is to merge the levels of a variable. An example is ethnicity, where the model did not converge when assessing for the “Black/Black British” and “Indian/Pakistani/Bangladeshi” subgroups in the Waves 2 to 3 dataset, separately or when merged. As a result, we merged both subgroups with the “mixed or other” ethnicity subgroup, limiting our analysis for ethnicity to white and non-white subgroups. Jackson discusses other causes of non-convergence and ways of handling them in the MSM framework [1].

# References

1. Jackson, C., *Multi-State Modelling With R: The MSM Package. 1.6. 1.6. 6.* Cambridge: School of Clinical Medicine, MRC Biostatistics, Unit, 2018.

2. Dinsdale, H., C. Ridler, and L. Ells, *A simple guide to classifying body mass index in children.* National Obesity Observatory: Oxford, 2011.

3. De Onis, M., *World health organization reference curves.* The ECOG’s eBook on child and adolescent Obesity, 2015. **19**.

4. Willekens, F. and H. Putter, *Software for multistate analysis.* Demographic Research, 2014. **31**: p. 381-420.

5. Willekens, F., *Continuous-time microsimulation in longitudinal analysis*, in *New frontiers in microsimulation modelling*. 2017, Routledge. p. 413-436.

6. Cooney, P. and A. White, *Extending Beyond Bagust and Beale: Fully Parametric Piecewise Exponential Models for Extrapolation of Survival Outcomes in Health Technology Assessment.* Value in Health, 2023. **26**(10): p. 1510-1517.

7. Brouwer, A.F., et al., *Transitions between cigarette, ENDS and dual use in adults in the PATH study (waves 1–4): multistate transition modelling accounting for complex survey design.* Tobacco control, 2022. **31**(3): p. 424-431.

8. Valliant, R. and J.A. Dever, *Survey weights: a step-by-step guide to calculation*. 2018: Stata Press College Station, TX.
